# Supplementary material for: Crowding in the Eye Lens: Modeling the Multisubunit Protein β-Crystallin with a Colloidal Approach
Source: Biophys J. 2020 Nov 13;119(12):2483–96. doi: 10.1016/j.bpj.2020.10.035 (PMC7822730; doi:10.1016/j.bpj.2020.10.035)
Supplement: Document S1 — . Supporting Materials and Methods and Figs. S1–S9 [file mmc1.pdf]

Biophysical Journal, Volume *119*

Supplemental Information

Crowding in the Eye Lens: Modeling the Multisubunit Protein β-Crystallin with a Colloidal Approach

Felix Roosen-Runge, Alessandro Gulotta, Saskia Bucciarelli, Lucía Casal-Dujat, Tommy Garting, Nicholas Skar-Gislinge, Marc Obiols-Rabasa, Bela Farago, Emanuela Zaccarelli, Peter Schurtenberger, and Anna Stradner

Biophysical *Journal*
Supporting Information

Supporting information: Crowding in the eye lens: modeling the multi-subunit protein β crystallin with a colloidal approach

Felix Roosen-Runge[1,*], Alessandro Gulotta[1], Saskia Bucciarelli[1], Lucía Casal-Dujat[1], Tommy Garting[1], Nicholas Skar-Gislinge[1], Marc Obiols-Rabasa[1], Bela Farago[2], Emanuela Zaccarelli[3,4], Peter Schurtenberger[1], and Anna Stradner[1,*]

[1]Division of Physical Chemistry, Lund University, Naturvetarvägen 14, 22100 Lund, Sweden
[2]Institut Laue-Langevin, 71 avenue des Martyrs, 38042 Grenoble, France
[3]Institute for Complex Systems, National Research Council (ISC-CNR), Uos Sapienza, Piazzale Aldo Moro 5, 00185 Rome, Italy
[4]Department of Physics, Sapienza Università di Roma, Piazzale Aldo Moro 1, 00185 Rome, Italy
[*]Correspondence: felix.roosen-runge@mau.se, anna.stradner@fkem1.lu.se

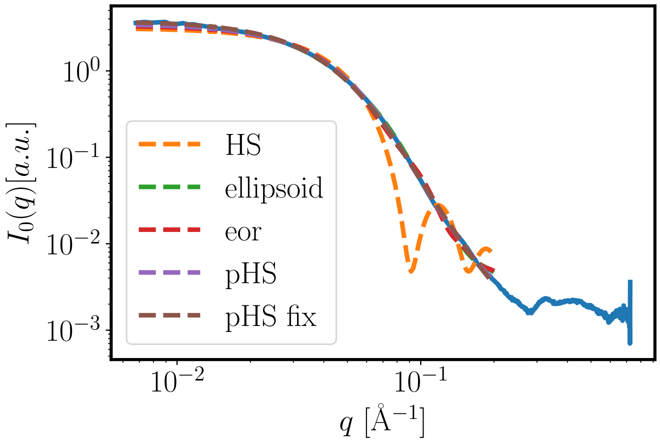

Figure S1: Different model fits (dashed lines) to the SAXS form factor of β_H crystallin (full line). While the hard sphere form factor (HS) cannot fit the data at all, form factors for a triaxial ellipsoid, an ellipsoid of revolution (eor) and polydisperse hard sphere systems provide reasonable fits. Given the evidence for polydisperse solutions, and the unfavorable R_h/R_g ratio for ellipsoids, we focused on the polydisperse hard sphere systems, and used both a fit with free parameter (pHS) and one with fixed radius of gyration (pHS fix; reported in main article).

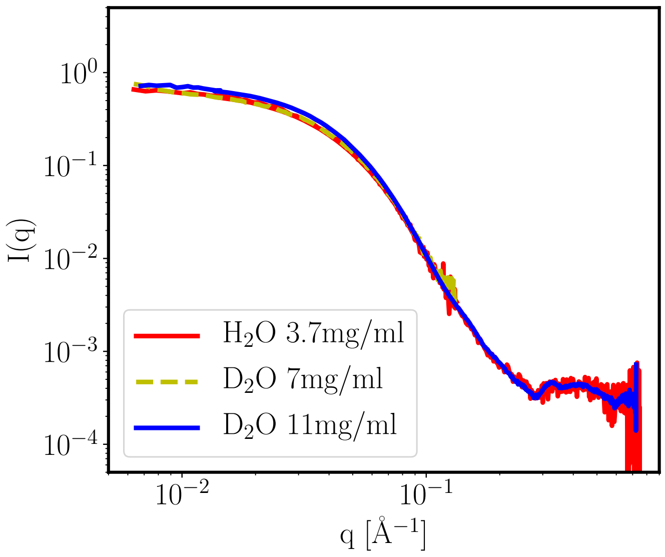

Figure S2: Form factor of β_H crystallin at different low protein concentrations measured by SAXS. All profiles in H_2O and D_2O show consistent results.

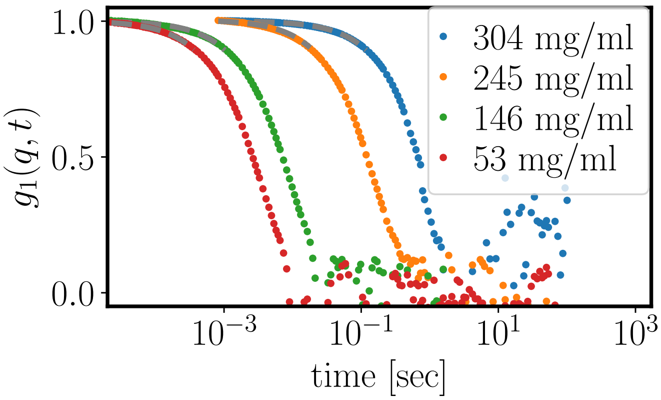

Figure S3: Exemplary correlation function from microrheology for tracer particles in concentrated β_H crystallin solutions. From the initial slope, the diffusion coefficient of the large tracers is calculated, which is directly related to the viscosity of the protein solution.

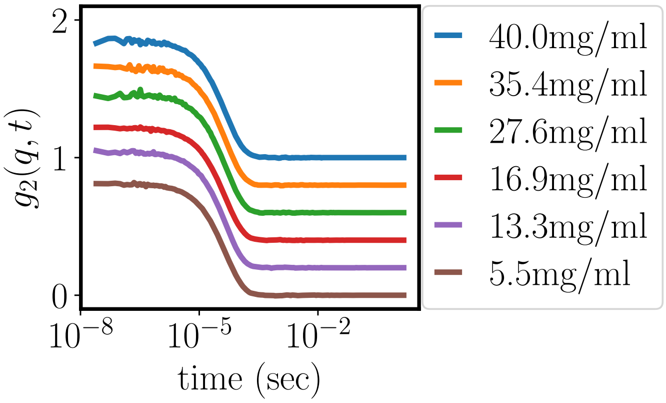

Figure S4: Exemplary correlation functions of β_H crystallin from DLS at a scattering angle 90° for lower concentrations. The data were shifted by 0.2 for better visibility, as the relaxation time is constant throughout the concentration range.

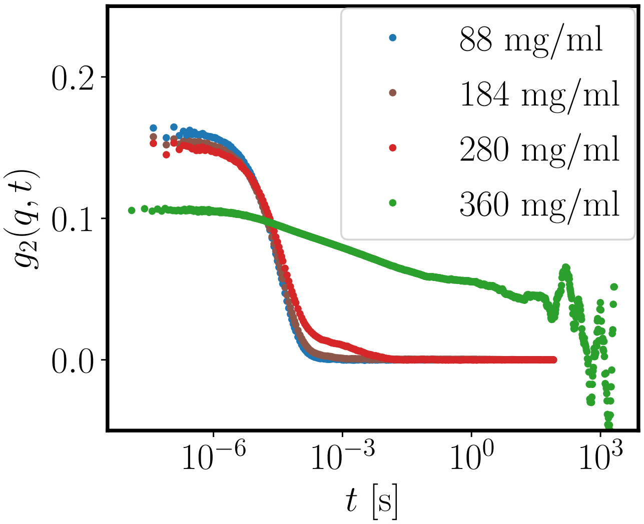

Figure S5: Autocorrelation functions of β_H crystallin from DLS at a scattering angle 90° for higher concentrations at q = 0.22 nm^1. While the three lower concentrations show reasonable profiles, the undefined baseline and the low intercept indicate non-ergodic sample properties for the highest protein concentration of 360 mg/ml. This profile was thus not fitted, but rather indicated a concentration where dynamical arrest has already set in.

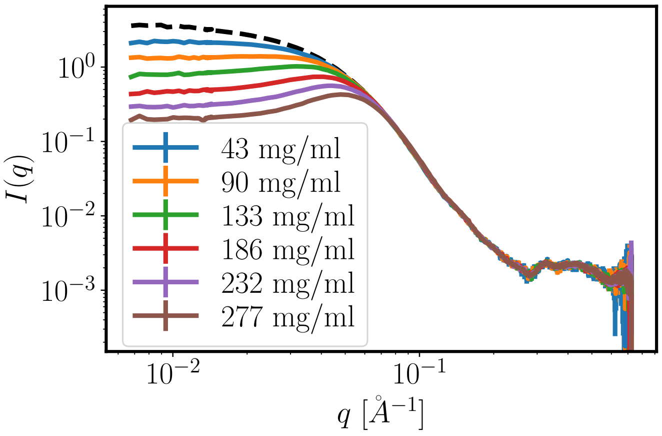

Figure S6: Normalized scattering intensities from SAXS for a concentration series of β_H crystallin. The form factor (dashed black line, 11 mg/ml) is used to calculate the experimental structure factor reported in the main article.

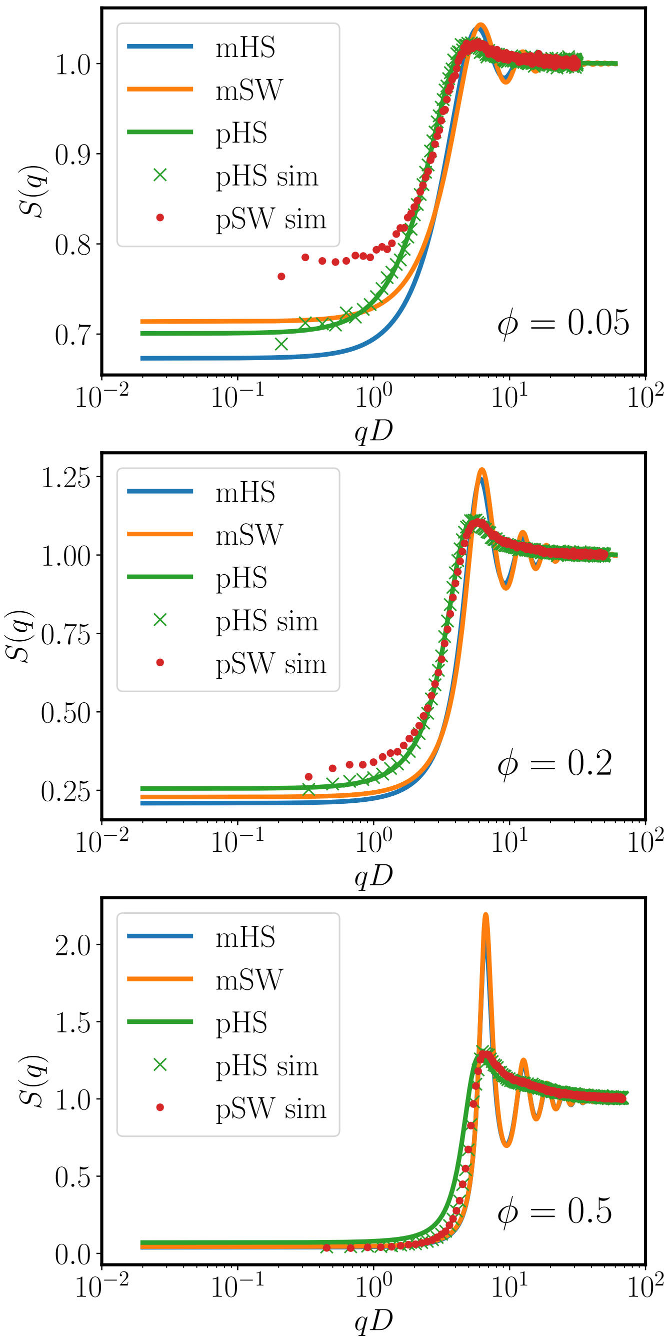

Figure S7: Effects of polydispersity and attraction on $S(q)$. The lines correspond to theoretical calculations of structure factors for monodisperse hard spheres (mHS), polydisperse hard spheres (pHS) and monodisperse hard spheres with additional square-well attraction (mSW). The symbols indicate simulation results of polydisperse hard spheres with and without additional square-well attraction.

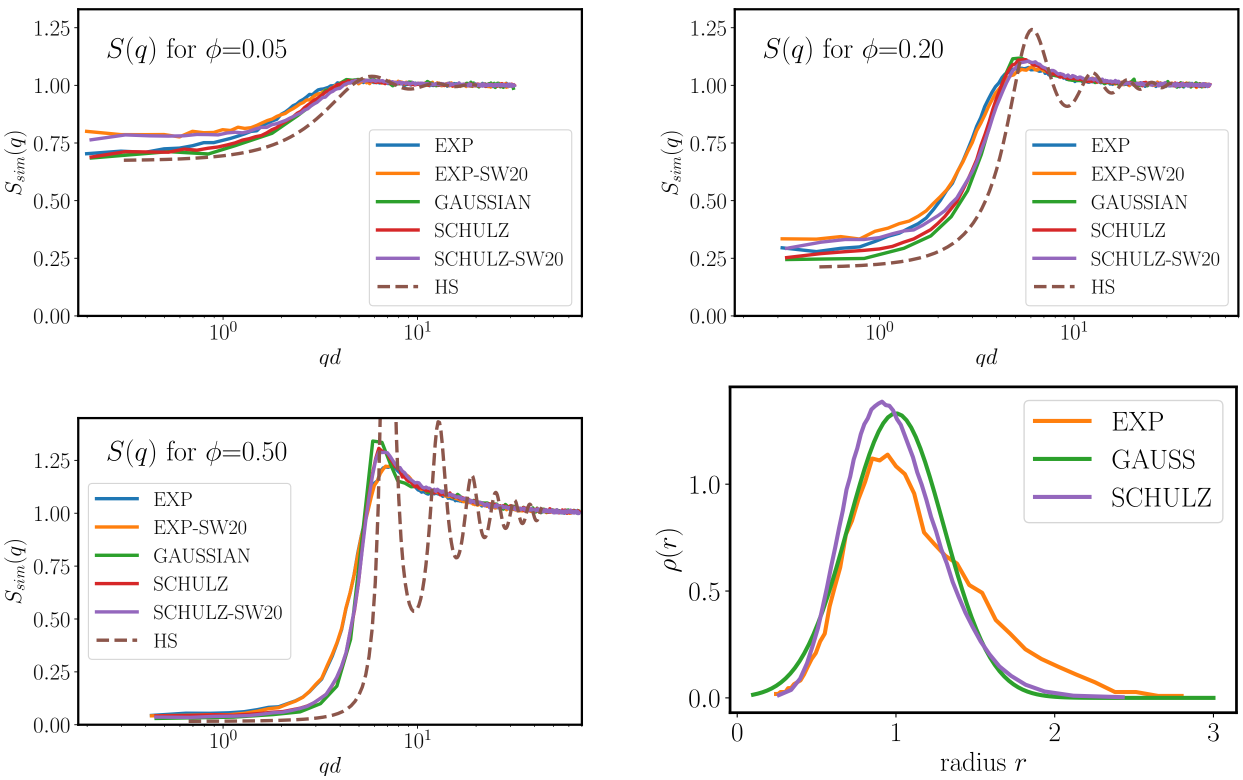

Figure S8: Effects of different size distributions (lower right) on the effective structure factor $S(q)$. The lines correspond to simulation of hard spheres with an exploratory size distribution (EXP), a Gaussian size distribution (GAUSS) and a discretized Schulz size distribution (SCHULZ), all with a normalized standard deviation of $\sigma^* = 0.3$. The discretized Schulz and the exploratory distribution were also simulated with an additional square-well attraction with a range of 0.2 of the diameter d and a depth of 0.289 k_BT (SW20). In addition, the structure factor of a monodisperse hard sphere is shown (HS).

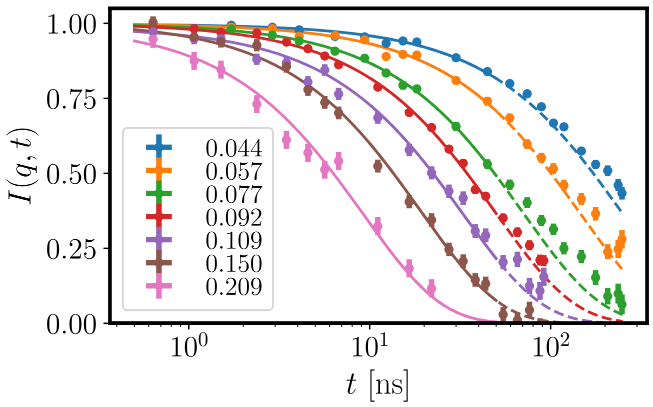

Figure S9: Intermediate scattering function $I(q,t)$ from neutron spin echo spectroscopy for β_H crystallin in D_2O phosphate buffer at a concentration of 88 mg/ml. The legend specifies the q value in Å^{-1}.

Exploratory size distribution

In addition to the methods used in the main text, we explored the use of inverse Monte Carlo methods for fitting of the form factor. We used the program package mcSAS (1, 2) which determines a distribution of hard spheres based on minimization of the experimental residuals, and additional regularization parameters to smoothen the distribution. As basic result, we obtained a distribution with a functional form very close to the Schulz distribution (see Fig. S8 lower right).

REFERENCES

1. Pauw, B. R., J. S. Pedersen, S. Tardif, M. Takata, and B. B. Iversen, 2013. Improvements and considerations for size distribution retrieval from small-angle scattering data by Monte Carlo methods. *Journal of Applied Crystallography* 46:365–371.

2. Bressler, I., B. R. Pauw, and A. F. Thünemann, 2015. *McSAS*: software for the retrieval of model parameter distributions from scattering patterns. *Journal of Applied Crystallography* 48:962–969.
