# Supplementary material for: Crowding in the Eye Lens: Modeling the Multisubunit Protein β-Crystallin with a Colloidal Approach
Source: Biophys J. 2020 Nov 13;119(12):2483–96. doi: 10.1016/j.bpj.2020.10.035 (PMC7822730; doi:10.1016/j.bpj.2020.10.035)
Supplement: Document S2. Article plus Supporting Material [file mmc2.pdf]

Biophysical Journal
Article

Biophysical Society

Crowding in the Eye Lens: Modeling the Multisubunit Protein β-Crystallin with a Colloidal Approach

Felix Roosen-Runge,[1,*] Alessandro Gulotta,[1] Saskia Bucciarelli,[1] Lucía Casal-Dujat,[1] Tommy Garting,[1] Nicholas Skar-Gislinge,[1] Marc Obiols-Rabasa,[1] Bela Farago,[2] Emanuela Zaccarelli,[3,4] Peter Schurtenberger,[1] and Anna Stradner[1,*]

[1]Division of Physical Chemistry, Lund University, Lund, Sweden; [2]Institut Laue-Langevin, Grenoble, France; [3]Institute for Complex Systems, National Research Council, Uos Sapienza, Rome, Italy; and [4]Department of Physics, Sapienza Università di Roma, Rome, Italy

ABSTRACT We present a multiscale characterization of aqueous solutions of the bovine eye lens protein β_H crystallin from dilute conditions up to dynamical arrest, combining dynamic light scattering, small-angle x-ray scattering, tracer-based microrheology, and neutron spin echo spectroscopy. We obtain a comprehensive explanation of the observed experimental signatures from a model of polydisperse hard spheres with additional weak attraction. In particular, the model predictions quantitatively describe the multiscale dynamical results from microscopic nanometer cage diffusion over mesoscopic micrometer gradient diffusion up to macroscopic viscosity. Based on a comparative discussion with results from other crystallin proteins, we suggest an interesting common pathway for dynamical arrest in all crystallin proteins, with potential implications for the understanding of crowding effects in the eye lens.

SIGNIFICANCE An increase in the concentration of protein solutions toward those found in living cells such as in the eye lens is often accompanied by a nonequilibrium arrest transition. The existence of such liquid-solid transitions has been linked with presbyopia, i.e., the eye lens' loss of accommodative capability with age. Here, we present that solutions of the multisubunit β_H crystallin, representing one of the three major lens protein classes, show such an arrest consistent with predictions for colloidal hard spheres. We uncover remarkably similar arrest scenarios for all crystallin classes, which is particularly surprising because the individual crystallins considerably differ both in their structural properties as well as in their equilibrium phase behavior. This observation will contribute to an improved understanding of presbyopia.

INTRODUCTION

The intracellular fluid within fiber cells in the eye lens is composed of a dense solution of mainly proteins from the crystallin family. This crowded solution with up to 400 mg/mL protein content has fascinating properties, ensuring not only a large enough refractive index, transparency, and flexibility of the lens required for visual function but also stability over the mammalian lifetime. Failure of solubility (e.g., protein condensation) and loss of flexibility result in conditions such as cataracts and presbyopia (1–3), which are leading causes for blindness and age-related vision problems.

To understand how macromolecular crowding can cause these very specific physicochemical properties of the eye lens, a complete picture of structural, dynamical, and thermodynamical consequences of crowding in protein solutions is required. Crowding affects a large range of properties, including inter alia structural stability, reaction equilibria, and long-range self-diffusion (4–6), but a conclusive picture on underlying mechanisms could not yet be obtained. This lack is linked to the necessity of obtaining a comprehensive multiscale picture to relate macroscopic phenomenology to microscopic mechanisms. In recent years, microscopic mechanisms were studied in more detail in model systems, outlining, e.g., the importance of self-association for resulting dynamical properties (7,8), the relevance of translational-rotational coupling (9), and the

Submitted June 18, 2020, and accepted for publication October 19, 2020.

*Correspondence: felix.roosen-runge@mau.se or anna.stradner@fkem1.lu.se

Editor: Jill Trewhella.

https://doi.org/10.1016/j.bpj.2020.10.035

© 2020 Biophysical Society.

This is an open access article under the CC BY license (http://creativecommons.org/licenses/by/4.0/).

relevance of hydrodynamic interactions for a quantitative understanding of protein diffusion (10–12). In this context, the explanatory power of colloid model systems for short-range diffusion in concentrated protein solutions proved successful (13,14) for a large range of globular proteins, including myoglobin (15), hemoglobin (16), ferritin (17), lysozyme (18,19), crystallin proteins (20,21), bovine serum albumin (11,22), and antibodies (7,8,12,23). However, only a few of these studies attempted to link to macroscopic dynamical and thermodynamical properties such as compressibility, viscosity, and dynamical arrest. The full potential of colloidal models has thus yet to be explored, in particular in situations of biological relevance in which the protein in question often shows a more complex behavior than conventional model systems.

In this context, crystallin proteins from the eye lens provide a promising test case to evaluate how a colloidal model can be used to mechanistically understand the uncommon physicochemical properties related to the eye's function. Based on size-exclusion chromatography, the crystallin proteins in the mammalian eye lens can be divided into three main classes: α-, β-, and γ-crystallin (Fig. 1; (24,25)). Previous publications focused on solutions of individual crystallin classes of the bovine eye lens, resulting in consistent colloidal pictures for both α- and γ_B crystallin, a subclass of bovine γ-crystallins. α-crystallin is known to occur in a large variety of compact oligomers (26) and can still be described by a polydisperse-hard-sphere system with average diameter around 15 nm regarding, e.g., the protein interaction, diffusion, and the repulsive glass transition (21,27–29). γ_B crystallin shows a rich dynamic and phase behavior with liquid-liquid phase separation, nonmonotonously varying diffusion, and dynamical arrest and can be understood as a slightly prolonged ellipsoid with a patchy attraction (20,21,30–33).

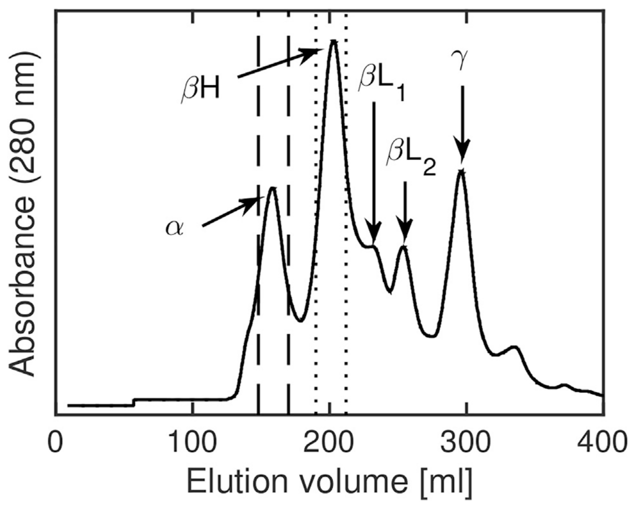

FIGURE 1 Elution absorption spectrum of a size-exclusion chromatography on the cortical extract of bovine lenses.

A similar description of bovine β-crystallin has not been obtained so far, as the complex structural properties present additional challenges. β-crystallin occurs in a broad range of smaller oligomers, observable as β_H, β_{L1}, and β_{L2} fractions in size-exclusion chromatography. Seven monomers of acidic ($\beta_{A1\text{–}4}$) and basic ($\beta_{B1\text{–}3}$) nature with molecular weights of 23–28 kDa (34) show a rather specific pairing interaction of monomers (35), resulting in a complex and potentially successive oligomerization into dimers, tetramers, and larger oligomers (25,36).

In this study, we focus on the main fraction of β-crystallin—the so-called $\beta_{\rm H}$ fraction—of larger polydisperse oligomers with molecular weights clearly beyond 100 kDa, including rather nonspecific oligomers of five to eight monomers with an open structure (24,36,37). We combine small-angle x-ray scattering (SAXS), dynamic light scattering (DLS), neutron spin echo (NSE), and tracer based microrheology with the final aim of obtaining a consistent picture based on a colloidal model on structural and dynamical properties of $\beta_{\rm H}$ solutions up to high volume fractions. With this combination of techniques, we obtain a multitechnique picture of dynamical arrest, which signifies the state of a sample in which large-scale motions freeze, resulting in solid-like, elastic properties of the sample, while small-scale motions such as rattling in the cage are still possible. Dynamical arrest should thus not be confused with an absolutely stationary system.

MATERIALS AND METHODS

Purification and sample preparation of $\beta_{\rm H}$ crystallin

Crystallin proteins were purified from calf lenses (byproducts from a slaughterhouse) using a well-established procedure (38,39). In this procedure, the eye lenses are ground to break the cell walls, and the resulting suspension is filtered and separated on a size-exclusion chromatography column (Hi-load Superdex 200 prep grade; Fig. 1) using 52.4 mM phosphate buffer (pH 7.1) as eluent phase, containing 1 mM dithiothreitol to prevent oxidation of the proteins and 0.02 wt% sodium azide to prevent bacterial growth. The $\beta_{\rm H}$ fraction was isolated and stored at low protein concentration in the same buffer. Amicon Ultra 10 kDa centrifuge filters were used for solvent exchange to the final measurement buffers and to reach the desired elevated concentrations.

As the final solvent, 52.4 mM phosphate buffer in H_2O and D_2O with 20 mM dithiothreitol and 0.02 wt% sodium azide at pH 7.1 was used for most experiments. Protein concentrations c_p were determined by ultraviolet absorption spectroscopy at a wavelength of $\lambda = 280$ nm using the specific absorption coefficient of β-crystallin, $E_{1\%,\ 280\ \rm nm} = 2.3$ mL mg^{-1}/cm (32).

To characterize a potential dynamical equilibrium of the oligomerization, we reanalyzed the collected β_H fraction using size-exclusion chromatography. Even after a waiting time of days, we obtain only a small amount of β_L oligomers in the collected β_H fraction, which we assign to the finite chromatographic resolution of the purification. Quantifying the mass ratios by peak integration, we obtain a mass ratio of β_L/β_H that is five times smaller than in the initial extract, which implies that the oligomerization cannot be considered as a dynamical equilibrium. Considering that the molecular weight of β_L oligomers is smaller by at least a factor of 2, the scattering contribution of these β_L oligomers is found to be below 3% of the

total scattering. This clearly indicates that the observed polydispersity is a generic property of β_H crystallin and not caused by the additional contributions to the scattering signal caused by the presence of β_L oligomers.

Dynamic light scattering

Dynamic light scattering (DLS) experiments of pure protein solutions were performed on two laboratory instruments: a goniometer system from ALV (Langen, Germany) and a home-built multiangle setup (40). All instruments are equipped with hardware cross-correlators, providing as data the average intensity $I(q)$ and the intensity-intensity correlation function $g_2(q, t)$. Here,

$$q = \frac{4\pi n}{\lambda} \sin(\theta / 2) \tag{1}$$

denotes the scattering vector for the scattering angle θ, the wavelength λ, and the refractive index $n = 1.33$.

We used the Siegert relation

$$g_2(q,t) = b + a g_1(q,t)^2 \tag{2}$$

to convert the intensity-intensity correlation function $g_2(q, t)$ to the field-field correlation function $g_1(q, t)$, which corresponds to the coherent intermediate scattering function $I(q, t)$. Here, b is a baseline term that usually equals 1 but can be slightly larger because of experimental artifacts. a denotes the speckle contrast factor. We used fit functions for $g_1(q, t)$ for direct fitting of $g_2(q, t)$ to be able to account for a and b in the same fitting step as the other parameters (41). As outlined later on, we used in particular cumulant analysis and double-decay functions with stretched exponential components. We estimated the error bars of $g_2(q, t)$ necessary for error-weighted fitting from the timewise standard deviation of three measured correlation functions on each sample.

Small-angle x-ray scattering

Small-angle x-ray scattering (SAXS) measurements on concentration series of the complete β_H crystallin fraction were performed on a laboratory instrument (Ganesha 300 XL SAXS System from SAXSLAB), which is a pinhole camera with a high brilliance microfocus sealed tube, a Pilatus detector, and a thermostatted sample stage. From the obtained two-dimensional detector images, an azimuthal average was performed, and the corresponding background was subtracted, resulting in the scattering profile $I(q)$, which is used to determine the form factor and experimental structure factor in this manuscript.

Neutron spin echo spectroscopy

Experiments were carried out on the neutron spin echo (NSE) spectrometer IN15 at the Institut Laue-Langevin in Grenoble, France. We used three different neutron wavelengths λ with three angles each to achieve the right q-values and maximal relaxation times τ_{max}: 1) $\lambda = 12.2$ Å at 11.14, 16.75, and 22.39° with $\tau_{max} = 91$ ns; 2) $\lambda = 22.8$ Å at 7.76, 10.87, and 15.55° with $\tau_{max} = 248$ ns; and 3) $\lambda = 22.8$ Å at 3, 6.24, and 10.41° with $\tau_{max} = 598$ ns.

These settings allow for a broad q-range from 0.25 to 2.1 nm^{-1}. After normalizing the raw echo intensities by the instrument resolution, we obtain the coherent intermediate scattering function $I(q, t)$, which was then used for further data analysis.

Microrheology

To measure the evolution of the zero-shear viscosity with increasing protein concentration, we performed microrheology based on DLS of tracer beads in the protein solution. This method allows us to obtain reliable results without using prohibitive amounts of concentrated protein solution (38). The basic idea of tracer-based microrheology based on DLS is to choose tracers much larger than the proteins so that bulk quantities of the protein solutions can be probed and the scattering signal is dominated by the tracers. From the obtained diffusion coefficient of the tracers, the viscosity of the surrounding protein solution can be calculated using the Stokes-Einstein formula. We used polystyrene particles with a diameter of 300 nm, sterically stabilized by a polyethylene glycol layer covalently bound to the particle surface, as characterized in detail in a previous study (42). We used a commercial goniometer system (three-dimensional DLS spectrometer from LS Instruments, Fribourg, Switzerland) at scattering angle $\theta = 90°$, allowing for suppression of multiple scattering using the modulated three-dimensional cross-correlation technology (43).

Molecular dynamics simulations

We used event-driven molecular dynamics simulations (44,45) to obtain a theoretical expectation of the structure factor in polydisperse and attractive hard spheres. At volume fractions ranging from 0.05 to 0.5, we used 2000 spheres with a discretized distribution of radii R_i with a polydispersity index of 0.3.

The basic distribution of the particle radius R is given by the so-called Schulz distribution

$$P(R) = \left(\frac{R}{b}\right)^{c-1} \frac{\exp(-R/b)}{b\Gamma(c)}, \tag{3}$$

where $c = 1/\sigma^{*2}$ is related to the normalized standard deviation σ^*, $b = \overline{R}/c$ is related to the mean radius $\overline{R}$, and $\Gamma(\cdot)$ denotes the gamma function. We stress that we use the Schulz distribution as a distribution of particle radius and not in the fundamentally different sense of polymer oligomerization.

The attraction between two particles i and j was represented by a square-well potential with a range of $0.25(R_i + R_j)$ and a depth of $u = 0.289 k_B T$. These parameters were chosen to have the same normalized second virial coefficient as the experimental one (see Eq. 14 below). The effective structure factor $S(q)$ was calculated directly from 300 independent configurations, taking into account the different form factors of individual particles. $S(0)$ was estimated from $S(q)$ using the average of the last five points in the low-q limit at which the data are roughly constant.

RESULTS

As a brief outline, we first used DLS and SAXS on dilute solutions to characterize the properties of an ensemble of protein molecules in the weakly to noninteracting regime. Second, we characterized the intermolecular interaction using DLS and SAXS. Third, we studied the emerging effects on dynamics in crowded and nearly arrested solutions reaching from the local level (NSE) over the density-gradient scale (DLS) to macroscopic relaxation (microrheology).

DLS: hydrodynamic radius and polydispersity

We used DLS under dilute conditions to obtain general information about the hydrodynamic size and polydispersity of the sample. To this end, we used the well-established second-order cumulant analysis (46) by fitting

$$\sqrt{g_2(q,t)-1} = ag_1(q,t) = a\exp\left(-t/\tau_0+\mu t^2/2\right) \quad (4)$$

Here, the prefactor $a = \sqrt{\sigma}$ is related to the contrast σ, $1/\tau_0$ as the first cumulant is the average relaxation rate, and μ is the second cumulant characterizing deviations from single exponential behavior. From these parameters, we obtain as a measure for the polydispersity the normalized standard deviation $\sigma^* = \sqrt{\mu}\tau_0$.

Fig. 2 (*top*) displays the correlation function for a solution with 2.26 mg/mL β_H crystallin in D_2O buffer (*symbols*) along with a second-order cumulant fit. Because this analysis neglects higher cumulants, the fit range in time has to be truncated at a suitable time. To allow a reasonable guess on this time, we systematically varied the truncation time and report the resulting parameters in Fig. 2 (*bottom*). As obvious from the graph, we obtain reliable and robust parameters around a reasonable truncation time of 0.1 ms, yielding a relaxation rate of $1/\tau_0 = 0.01592$/s and a σ^* of 0.30. Given the scattering vector $q = 0.023$ nm^{-1} with the refractive index $n = 1.33$, we obtain a diffusion coefficient of $D_0 = 1/(\tau_0 q^2) = 3.04 \times 10^{-11}$ m^2/s for β_H crystallin in D_2O phosphate buffer at $T = 25°C$.

The obtained z-averaged hydrodynamic radius reads from the Stokes-Einstein relation

$$R_h = \frac{k_B T}{6\pi\eta D_0} = 6.5 \text{ nm}, \quad (5)$$

where k_B is the Boltzmann constant, T is temperature, and η is the solvent viscosity.

Form factor from SAXS: overall shape and polydisperse modeling

The scattering intensity $I_0(q)$ measured in a dilute solution of β_H crystallin ($c_0 = 11$ mg/mL) provides access to the overall shape of the protein via the form factor (Fig. 3). Guinier analysis of the low-q data results in a radius of gyration $R_g = 4.8$ nm. As a first indicator of the overall shape, we obtain the ratio $R_h/R_g = 1.35$ (cf. *gray area* in Fig. 4). We remark that form factors at lower concentration both in H_2O and D_2O buffer show similar results (cf. Supporting

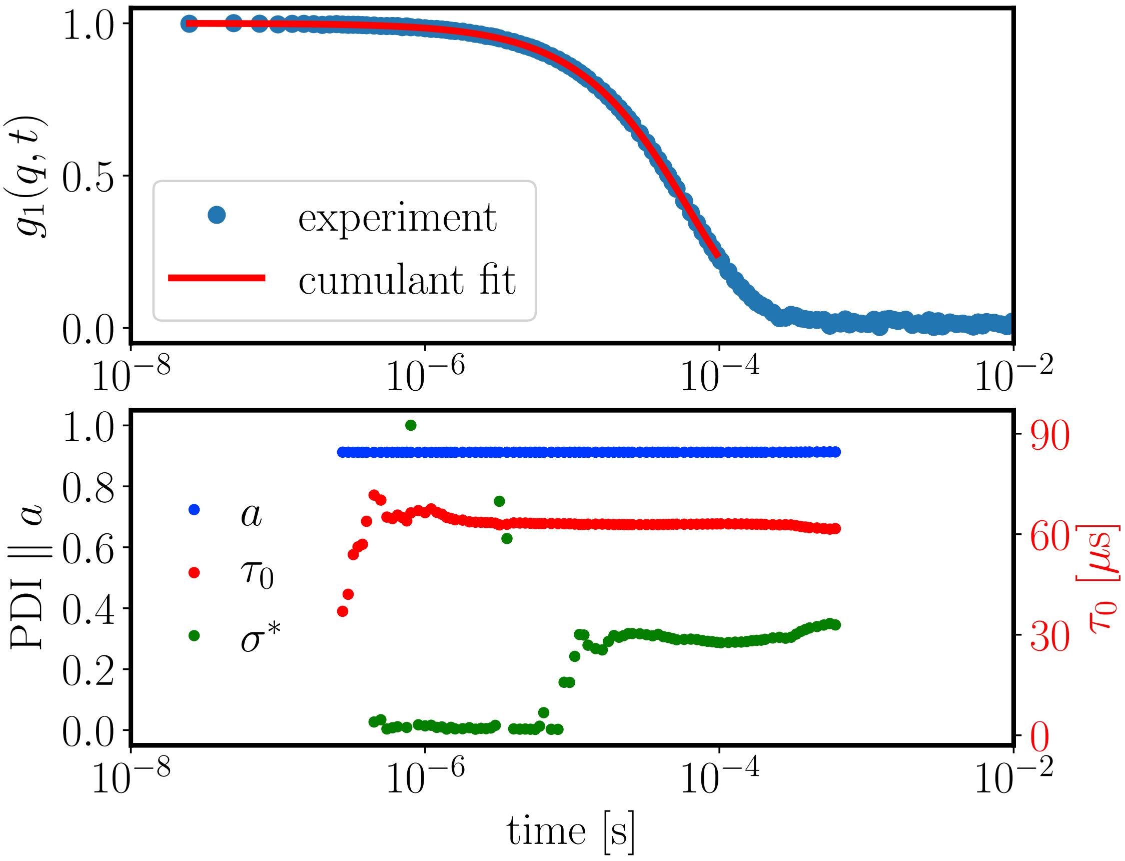

FIGURE 2 Cumulant analysis of $g_1(q, t)$ for a solution with 2.26 mg/mL concentration of β_H crystallin in D_2O phosphate buffer at 25°C. Top: $g_1(q, t)$ for scattering angle 120° along with the chosen fit for a truncation time of 0.096 ms is shown. Bottom: sensitivity analysis regarding the truncation time is given, showing a clean plateau around 0.1 ms with a radius distribution with normalized standard deviation $\sigma^* = 0.3$. We remark that the stable values for a and τ_0 indicate a robust fitting of the overall profile, which allows us to focus on σ^*. To see this figure in color, go online.

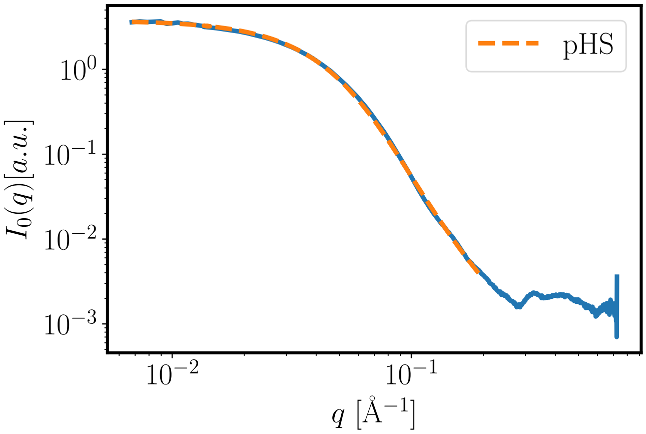

FIGURE 3 Scattering intensity $I_0(q)$ for a β_H crystallin solution at a dilute concentration of 11 mg/mL (*blue*). The form factor fitted using a polydisperse-hard-sphere system with radii distributed according to a Schulz distribution (pHS, *orange*) agrees well with the experiments on the fitted range up to 0.2 Å^{-1}. Fit parameters are a normalized standard deviation σ^* of 0.48 and an average radius (number average) of $\overline{R} = 2.51$ nm, which corresponds to an $R_h = 5.35$ nm and an $R_g = 4.8$ nm via Eq. 7. Experimental error bars are smaller than the line thickness. To see this figure in color, go online.

Materials and Methods) but face larger systematic errors due to background subtraction. For this reason, we used the form factor from a concentration of 11 mg/mL for further analysis.

As comparison, we calculated the theoretical values for polydisperse and hard ellipsoids of revolution (Fig. 4). To this end, we used the analytical expressions for R_h (47) and

$$R_g = b\sqrt{\frac{2+p^2}{5}} \tag{6}$$

as a function of the aspect ratio p, here defined as the polar axis a over the equatorial axis b.

From this model calculations, we obtain two qualitative results: first, nonsphericity induces smaller ratios compared to the maximum of $R_h/R_g = \sqrt{5/3} \approx 1.29$ for hard spheres with aspect ratio 1. Second, polydispersity with a σ^* beyond 0.1 also causes a significant decrease of the ratio.

We remark that we calculate the intensity-averaged radii to obtain the correct weighting for experimental observables from scattering:

$$R_h = \langle R^6 \rangle / \langle R^5 \rangle = \langle R \rangle (\sigma^*)^2 \left(5 + \frac{1}{\sigma^{*2}}\right) \tag{7}$$

and

$$R_g = \sqrt{\frac{3\langle R^8 \rangle}{5\langle R^6 \rangle}} = \langle R \rangle (\sigma^*)^2 \sqrt{\frac{3}{5}\left(7 + \frac{1}{\sigma^{*2}}\right)\left(6 + \frac{1}{\sigma^{*2}}\right)}, \tag{8}$$

where we used the fact that the nth moment of the Schulz size distribution reads

$$\langle R^n \rangle = \langle R \rangle^n (\sigma^*)^{2n} \frac{\Gamma[n + 1/\sigma^{*2}]}{\Gamma[1/\sigma^{*2}]} \tag{9}$$

From this analysis, we can conclude that an additional structural property of β_H crystallin causes a larger hydrodynamic size as expected for compact, smooth objects. Given the multisubunit character of β_H crystallin, a corrugated surface is likely and might be the cause of this signature. We remark that a protein assembly with a dense shell and more open core, as reported for other multisubunit proteins such as viruses and also α_B crystallin (26), would result in opposite effects and is thus not consistent with the ratio R_h/R_g of β_H crystallin.

From DLS, we have clear indications for a polydisperse nature with a large σ^* around 0.3, which implies a ratio below 1.2 for assumed smooth particles. Although a definite conclusion is not possible from this analysis, a further significant decrease due to nonsphericity seems unlikely because it would have to be compensated by an even stronger corrugation profile.

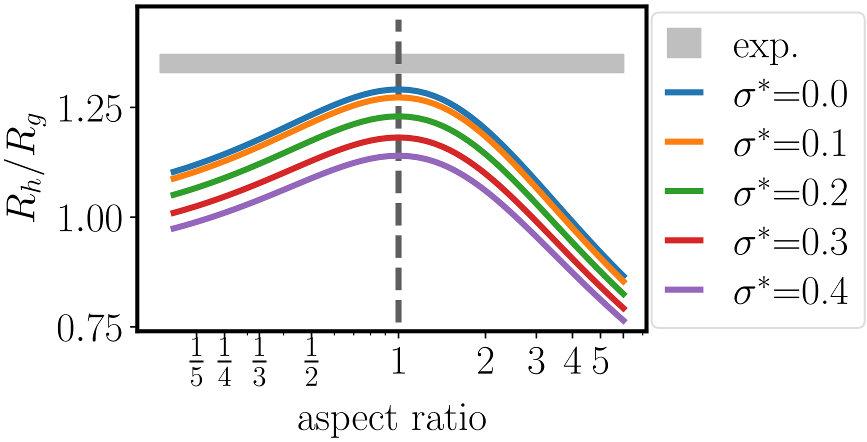

FIGURE 4 Ratio of hydrodynamic radius R_h over radius of gyration R_g for polydisperse spheres and polydisperse ellipsoids of revolution (*lines*) compared with the experimentally determined value (*gray area*). The aspect ratio p is the ratio of polar versus equatorial axis, i.e., $p > 1$ are prolate and $p < 1$ are oblate spheroids. For the polydispersity, we assumed a Schulz distribution for the half-axes with average radius 1 and varying normalized standard deviation σ^*. To see this figure in color, go online.

As a first check for a potential descriptive model based on polydisperse spheres, we fitted the form factor with a polydisperse-hard-sphere system with Schulz-distributed radii. To make the fit more robust, we constrained the fit parameters of the Schulz distribution to the experimentally observed $R_g = 4.8$ nm, i.e., we set the number-average radius via Eq. 8, and only fitted $\sigma*$, a constant background, and a scalar prefactor. The fit result is in good agreement with the experimental form factor on the fitted q-range up to 0.2 $Å^{-1}$ (Fig. 3).

As with the fitted $\sigma*$, we obtain again a relatively large value of $\sigma* = 0.475 \pm 0.003$, which supports our picture of a highly polydisperse system. We remark that the features at larger q-values are related to the internal structures of β_H crystallin, which are not the focus of this study.

Further model fits are shown in the Supporting Material. We remark that reasonable model fits can also be obtained by monodisperse ellipsoids, but elongated and oblate shapes would induce a large discrepancy in the ratio R_h/R_g (Fig. 4).

DLS: constant gradient diffusion at intermediate concentrations

Fig. 5 shows normalized diffusion coefficients of concentration series of β_H crystallin in D_2O and H_2O phosphate buffer at three temperatures. A set of correlation functions is shown in the Supporting Material. To obtain a robust measure, we used single exponential fits to $g_2(q, t)$:

$$g_2(q,t) = b + a\exp(-2t/\tau_1), \tag{10}$$

where a is the intercept, b is a constant background, and τ_1 is the relaxation time, which is related to the diffusion coefficient D by

$$\frac{1}{\tau_1} = Dq^2 \tag{11}$$

Interestingly, the diffusion coefficients are remarkably constant, implying that an increased protein concentration does not vary the relaxation of large-scale concentration gradients. In terms of conventional light scattering notation (48),

$$D = D_0(1 + K_d c), \tag{12}$$

we obtain a diffusion parameter $K_d \approx 0$.

This behavior points toward a partial compensation of attraction and repulsion: purely repulsive particles generally experience an enhanced gradient diffusion at higher concentration with $K_d > 0$, whereas attraction induces a slowing-down of gradient diffusion with increasing concentration, resulting in $K_d < 0$ for sufficient attraction strength.

This general consideration can be quantified based on theoretical calculations. For gradient diffusion of sticky hard spheres including hydrodynamic interactions, one obtains (49)

$$K_d = (1.454 - 1.125/\tau)\nu_{\text{eff}}, \tag{13}$$

with the effective voluminosity ν_{eff} of the protein and the stickiness parameter τ. The finding $K_d \approx 0$ has two important consequences: first, we can obtain an estimate on the interaction without assuming a voluminosity ν_{eff} because of the factorization of the right-hand term. Second, the corresponding stickiness $\tau \approx 0.774$ can be translated to a normalized second virial coefficient $B_2^{(SHS)}$ with (50,51)

$$B_2^{(SHS)} \Big/ B_2^{(HS)} = 1 - \frac{1}{4\tau} \approx 0.677, \tag{14}$$

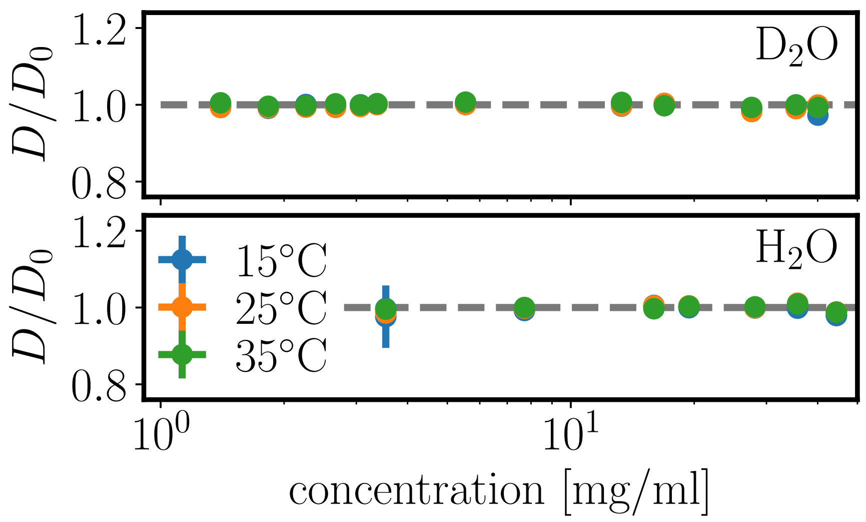

FIGURE 5 Gradient diffusion coefficients for a concentration series of β_H crystallin. No significant deviation from a constant value is observed up to relatively large concentrations. We remark that D and D_0 depend on the temperature and viscosity in the same way, and the ratio D/D_0 thus is indicative of effects beyond those expected because of changes of temperature or solvent isotope. Experimental error bars are shown, but mostly smaller than the symbol size. To see this figure in color, go online.

where $B_2^{(HS)} = 4V$ denotes the virial coefficient of hard spheres with volume V. The ratio $B_2^{(SHS)}/B_2^{(HS)}$ in rough terms indicates that one-third of the hard-core repulsion is compensated by weak attraction.

We stress that K_d, in particular for $K_d \approx 0$, is a very sensitive measure of protein interaction and thus provides a reliable test for changes of interaction due to temperature and isotopes. The finding of a nonobservable effect of temperature and solvent isotopes on the interaction, as visible from Fig. 5, is an important finding for β_H crystallin and not generally expected. Other protein systems often show clear isotope effects on phase behavior and protein interactions when exchanging H_2O to D_2O (51–53). The missing isotope effect in β_H crystallin demonstrates that the attractive interaction is generic for the protein, and not induced by a specific solvent condition. Importantly, this finding also suggests that the similarly observed characteristics in H_2O and D_2O phosphate buffer are also relevant for physiological conditions.

We remark that a constant D/D_0 over a broad concentration range could, in principle, be induced by an equilibrium between different oligomeric species. We judge that this picture is unlikely for three main reasons: first, for an equilibrium, a Boltzmann factor should govern the oligomer ratios, and one would thus expect an effect of temperature, at least for enthalpic contributions. The absence of a temperature effect thus implies no significant association enthalpy. Second, a potential entropic contribution would usually be linked to water-mediated interaction. The absence of a solvent isotope effect thus implies a negligible association entropy. Third, such a constant profile would be very coincidental because mass action involves different terms with different concentration dependence that would need to cancel completely.

Slow relaxation: microrheology and slow mode from DLS

When moving toward concentrations higher than 50 mg/mL, the correlation function picture becomes more complex (Fig. 6). With increasing concentration, a significant second relaxation mode is observed. Using a fit model with two stretched exponentials

$$g_2(q,t) = b + a\left(c\exp[-(t/\tau_1)^{\alpha_1}] + (1-c)\exp[-(t/\tau_2)^{\alpha_2}]\right)^2, \quad (15)$$

we quantify the average relaxation time of the slow stretched exponential as

$$\overline{\tau} = \frac{\tau_2}{\alpha_2}\Gamma\left[\frac{1}{\alpha_2}\right] \quad (16)$$

After normalization with the relaxation time of an imaginary dilute system $\tau_0 = 1/D_0q^2$, we show the resulting re-

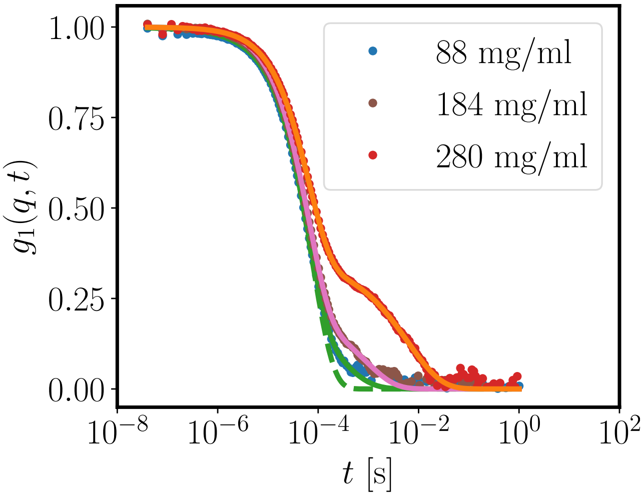

FIGURE 6 Correlation functions $g_1(q, t)$ for different concentrations of β_H crystallin in D_2O buffer at $q = 0.22$ nm^{-1}. Solid lines correspond to fits with double stretched exponentials to the correlation functions for protein concentrations of 88, 184, and 280 mg/mL measured with the multiangle DLS instrument (angle 90°). The dashed line denotes a cumulant fit, indicating a contribution of further decays already at comparably low concentrations. To see this figure in color, go online.

scaled $\overline{\tau}/\tau_0$ in Fig. 7 (*orange circles*). The corresponding stretching exponents α_2 are 0.59, 0.75, and 0.66 for the samples with 88, 184, and 280 mg/mL protein concentration, whereas the fits yield α_1 very close to 1 in all cases.

We remark that for the highest measured concentration of 360 mg/mL in D_2O buffer, both the low intercept and the undefined baseline suggest a potentially nonergodic sample because of the onset of dynamical arrest (see Fig. S5). We

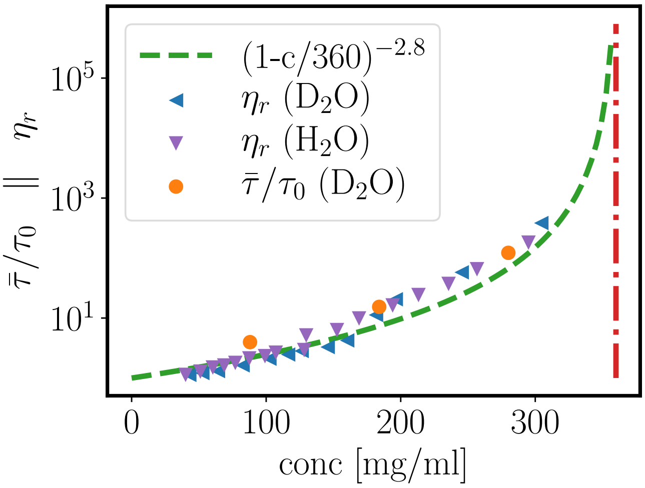

FIGURE 7 Relative viscosity η_r of β_H crystallin in H_2O (*downwards triangles*) and D_2O (*sidewards triangles*) phosphate buffer and normalized relaxation time $\overline{\tau}/\tau_0$ from the slow relaxation mode in DLS (*circles*) agree well with a power-law scaling (*dashed line*). The dash-dotted line indicates a sample with a nonergodic signature in the observed relaxation, indicating proximity to dynamical arrest. The viscosity data of β_H crystallin in H_2O buffer are reproduced from (42). To see this figure in color, go online.

thus indicate this concentration as a concentration close to arrest with a red vertical dash-dotted line.

As a second technique exploring the slow relaxation in concentrated solutions of β_H crystallin, we use DLS-tracer microrheology to obtain the relative viscosity

$$\eta_r = \frac{\eta_0}{\eta_s}, \tag{17}$$

where η_0 is the zero-shear viscosity of the sample and η_s is the solvent viscosity.

We obtain good agreement of the viscosity with $\overline{\tau}/\tau_0$, implying that the slow relaxation of gradient diffusion governs the viscosity property of β_H crystallin solutions. The broken line represents a power-law scaling for the relative viscosity:

$$\eta_r = \left(1 - \frac{c}{c^*}\right)^{-\gamma}, \tag{18}$$

with $c^* = 360$ mg/mL and $\gamma = 2.8$. Compared with mode-coupling theory and computer simulations, the scaling exponent $\gamma = 2.8$ is consistent with a system close to hard spheres, whereas for significantly increased attraction, one would expect a larger exponent $\gamma > 3$ (54,55).

Voluminosity of β_H crystallin

Before moving on to a more detailed characterization of interactions and local dynamics, it is worth discussing the choice of an appropriate concentration variable that will allow us to explore analogies with colloids. So far, we have discussed all our findings as a function of the weight concentration of the protein. However, when using colloid theory, we need to switch to volume fractions as the relevant control parameter. The link between weight concentration and volume fraction is commonly made by using an effective voluminosity ν_{eff}. In contrast to the specific volume of the protein, which is typically on the order of 0.7–0.75 mL/g and basically determined by the volume of the individual amino acids in the protein, ν_{eff} corresponds to the volume from which other proteins are excluded. It thus corresponds to the volume of the structure defined by the surface encompassing the three-dimensional protein structure. Voids within a multisubunit protein or corrugated surfaces will thus lead to a value that can be significantly larger than the specific volume of the protein. Here, we have employed two different approaches to obtain a reliable estimate of ν_{eff}.

First, we focused on the packing at large protein concentrations. The arrest for systems of spheres with only mild attraction should occur at a volume fraction around 0.64, which in combination with an estimated experimental arrest concentration around 360 mg/mL amounts to a first estimation for the voluminosity of 0.64/(360 mg/mL) = 1.78 mL/g.

Second, we used the hydrodynamic radius, i.e., a measure based on the dilute concentration range. The hydrodynamic radius in a polydisperse system denotes the z-average, i.e., $R_h = \langle R^6 \rangle / \langle R^5 \rangle$. Using a Schulz distribution with $\sigma^* = 0.3$, one obtains a corresponding average volume of the polydisperse spheres of $V = 4\pi\langle R^3 \rangle/3 = 489.8$ nm^3. Conventional estimates for the average molecular weight of β_H crystallin are around $M_w = 180$ kDa (36,37). Dividing both values, we obtain as a second estimation a voluminosity of 1.64 mL/g.

We stress that the discrepancy between the two values does not imply an inconsistent data set but is caused by the different physical quantity used for the estimation. The first estimation is based on dense packing and governed by steric interaction, whereas the second estimation employs the hydrodynamic friction at low concentrations.

For the further analysis, we thus opt for an intermediate value for the effective voluminosity of $\nu_{\mathrm{eff}} = 1.7$ mL/g to compare experimental results on a broad concentration range to theoretical predictions and simulations. We remark that this value is much larger than the specific volume of proteins around 0.71–0.74 mL/g, which implies that the formed oligomeric structures incorporate a considerable volume of water, which is accounted for in the effective voluminosity ν_{eff}.

Although water in the inside of oligomers affects the static and dynamic estimation of the voluminosity similarly, the amount of water at the outer surface of the oligomer is different; whereas the static picture accounts for additional water filling the gaps between irregular oligomer surfaces in dense packings, the dynamic estimation is based on the water that is dragged along with the oligomers in dilute conditions. Within this picture, it is interesting to see that the extra amount of water in dense packing is larger than the water dragged along with the oligomer, and thus, some part of the water in dense packings might be less affected by movements of oligomers.

Forward scattering: isothermal compressibility

To characterize the overall thermodynamics of the protein solution, we measure the forward scattering intensity $I(q \to 0)$. Practically, we used the experimental structure factor

$$S(q) = \frac{I(q)}{I_0(q)} \frac{c_0}{c_p}, \tag{19}$$

where c_0 and c_p are the protein concentrations in the noninteracting solution (form factor) and interacting solutions. The low-q limit $S(q \to 0)$ was calculated as an average over the lowest points in q and is linked to the isothermal compressibility.

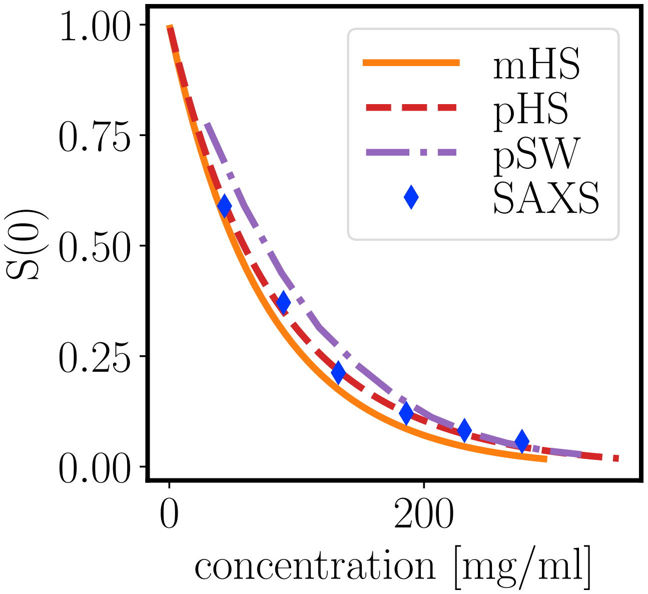

FIGURE 8 Forward scattering intensity $S(0)$ from SAXS. For comparison, the theoretical predictions for monodisperse hard spheres (mHSs) and polydisperse hard spheres (pHSs) are shown. Furthermore, simulation results for polydisperse hard spheres with an additional square-well attraction of $u/k_BT = 0.289$ (pSW) are shown. The size distribution for the simulation and theory correspond to a Schulz distribution with $\sigma^* = 0.3$. The theoretical values are scaled according to an effective voluminosity of $v_{\mathrm{eff}} = 1.7$ mL/g. To see this figure in color, go online.

Fig. 8 shows the data obtained from SAXS (*symbols*). As expected for an overall repulsive system, $S(0)$ decreases with increasing concentration, as also reported and discussed previously (32).

To compare this decrease to theoretical predictions, we calculated $S(0)$ for different model systems, always using an effective voluminosity $v_{\mathrm{eff}} = 1.7$ mL/g. First, we compared results with monodisperse spheres based on the Carnahan-Starling prediction (monodisperse hard spheres, *orange line*):

$$S(0) = \frac{(1-\phi)^4}{(1+2\phi)^2 + \phi^3(\phi - 4)} \tag{20}$$

Second, we used the theoretical solution for polydisperse hard spheres with Schulz-distributed radii (56) with a σ^* of 0.3 to account for effects of polydispersity (polydisperse hard spheres (pHSs), *red dashed line*). Finally, we used molecular dynamics simulations of polydisperse hard spheres with an additional square-well attraction (pSW, *purple dash-dotted line*). As general trends, polydispersity mildly increases the $S(0)$-values, whereas attraction causes significant increase.

With the voluminosity of $v_{\mathrm{eff}} = 1.7$ mL/g, we obtain agreement with the data for polydisperse hard spheres without attraction. We remark that one would need to use a very high voluminosity beyond 2 mL/g to reproduce the $S(0)$ profile with the pSW simulation. This implies that we cannot fully reproduce the trend of isothermal compressibility because this appears to be closer to the hard-sphere limit than to the expected curve with the mild attraction. This discrepancy requires more investigation using a structurally more refined model.

Structure factor

Having characterized the protein interactions from the perspective of density relaxations from DLS and isothermal compressibility from forward scattering, we complemented the long-range picture with local information from the experimental structure factor inspired by colloids (see Eq. 19).

Fig. 9 *a* summarizes the experimental structure factors. Whereas the low-q intensity evidences an overall repulsive system, the correlation peak at intermediate q-values is only weakly established and rather broad even for the high concentrations.

Fig. 9 *b* shows effective structure factors from a molecular dynamics simulation for a polydisperse system of hard spheres with additional attractive square-well potential (*solid lines*). For comparison, we also plot the theoretical structure factor for monodisperse hard spheres with attraction for volume fractions 0.05, 0.2, and 0.5 (*dashed lines*) (57). We have furthermore obtained similar results for different size distributions and attraction (cf. Supporting Materials and Methods). As a general result, polydispersity significantly reduces the peak height of the principal peak compared with pure hard spheres, which can be understood as polydisperse systems do not have a single preferred neighbor distance. We remark that polydispersity at the same time only mildly affects the low q (cf. previous section on forward scattering). In contrast, short-range attraction only has a minor effect on the peak height but significantly increases the low-q region. Although we obtained a strong and systematic decrease of the peak value for polydisperse systems compared with monodisperse hard spheres in simulations and theoretical predictions, the experimental absolute peak values are not reached for physically reasonable parameters within this model.

As an additional unusual feature, the shape of the experimental $S(q)$ has the feature of a moving shoulder at low q, whereas the simulations rather suggest a quasi-isosbestic point.

The colloidal model of polydisperse mildly attractive hard spheres with Schulz size distribution thus cannot fully reproduce the obtained local structure in crowded solutions of β_{H} crystallin. Given the likely rather corrugated multisubunit structure of β_{H} crystallin, these results can be linked to a broader range of nearest neighbor distances, which inevitably will decrease the peak height and increase the width of the principal peak that characterizes the regularity of the

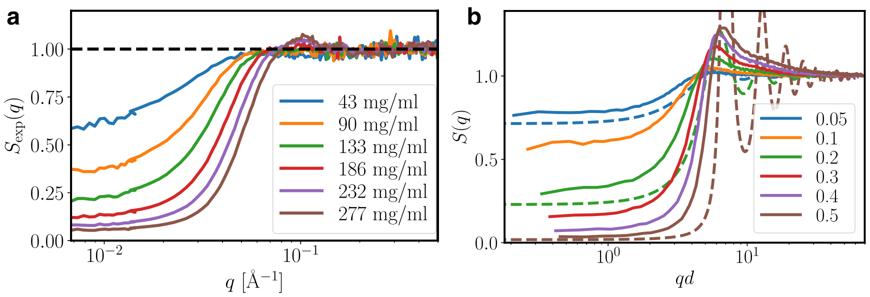

FIGURE 9 (*a*) Experimental structure factors from SAXS measurements for a concentration series of β_H crystallin in D_2O phosphate buffer. (*b*) Effective structure factors from simulations for a volume fraction series (as specified in the legend) of polydisperse spheres with short-ranged square-well attraction with depth $u = 0.289 k_B T$ and diameter d are shown. With the voluminosity $\nu = 1.7$ mL/g, the volume fraction range corresponds to a concentration range from 29.4 mg/mL ($\phi = 0.05$) over 118 mg/mL ($\phi = 0.2$) to 294 mg/mL ($\phi = 0.5$). The dashed lines indicate the structure factor for monodisperse spheres with the same square-well interaction at volume fractions 0.05, 0.2, and 0.5. To see this figure in color, go online.

local packing. We remark that a complete quantitative agreement on this local scale cannot be expected with such a simple model but that the good agreement of the overall qualitative picture is consistent.

NSE: short-time self- and cage diffusion

NSE spectroscopy provides access to diffusive motions on the local scale of the macromolecules, i.e., few nanometers. On these macromolecular scales, different types of diffusion occur simultaneously in the sample. First, proteins perform translational self-diffusion, i.e., Brownian motion. Second, proteins have rotational and internal degrees of motional freedom, resulting in contributions of rotational diffusion and interdomain motion. Third, density relaxations of macromolecules on length scales of $2\pi/q$ defined by the scattering vector q are often represented by the diffusion function $D(q)$. This q-dependent diffusion coefficient can directly be related to the structural correlations in the system, as the additional q^2 dependence of the relaxation time characteristic for diffusive motion is removed. Importantly, $D(q)$ roughly scales as $1/S(q)$ (58), which implies that strong spatial correlations relax more slowly because of the structural underpinnings. In this context, the principal peak q^* of the structure factor $S(q)$ represents an important case, being referred to as cage diffusion $D(q^*)$. Cage diffusion characterizes the escape of proteins from local arrangements of a neighbor cage in more concentrated solutions.

Fig. 10 shows the intermediate scattering functions for a concentration series of β_H crystallin at $q = 0.0566$ Å^{-1}, evidencing excellent statistics for all concentrations (for a data set with varying q, cf. Supporting Materials and Methods). To obtain a robust measure of the relaxation times τ_s, we fitted the initial slope (correlation time $<$ 50 ns) of the obtained intermediate scattering functions. From this, the diffusion function $D(q) = 1/(\tau_s q^2)$ can be calculated.

Fig. 11 displays $D(q)$ for a concentration series of β_H crystallin. As expected, the diffusion coefficients decrease with increasing protein concentration. Furthermore, the diffusion function is constant at scattering vectors larger than 0.05 Å^{-1} for higher concentrations. The observed increase at lower concentrations might be due to rotational diffusion and internal dynamics, which add their contribution to the underlying translational diffusion at larger q (13,59).

The contribution of internal and rotational diffusion indeed becomes apparent, when extracting the crowding dependence from the diffusion coefficients (Fig. 12). An extrapolation of both diffusion profiles to dilute concentration exceeds the dilute limit from DLS, which consistent with earlier results (11,13,59) suggests that, on top of the translational self-diffusion, additional contributions from rotational and internal motions are present. The cage diffusion was extracted around the principal peak q^* observed in $S(q)$ (the q^*-range used is indicated as a *blue bar* in Fig. 11). The cage diffusion shows slightly smaller diffusion coefficients, which most likely is due to the lower amount of rotational and internal contributions at q^* because no significant modulation of $D(q)$ is expected because of the weak correlation peak in $S(q)$.

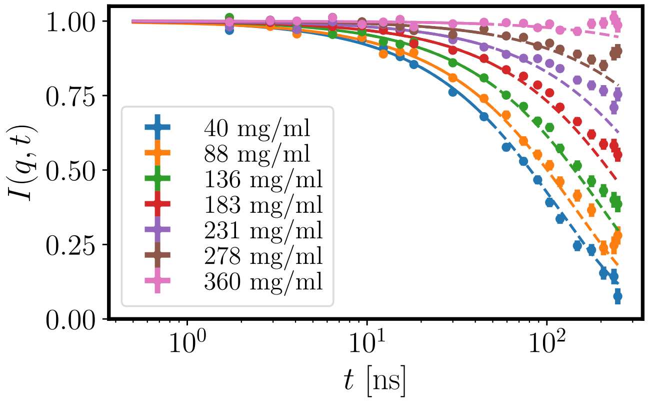

FIGURE 10 Correlation functions from NSE at $q = 0.0566$ Å^{-1} for a concentration series of β_H crystallin in D_2O phosphate buffer. The lines represent single exponential fits, and the solid line indicates the fit range $t < 50$ ns for the initial slope. To see this figure in color, go online.

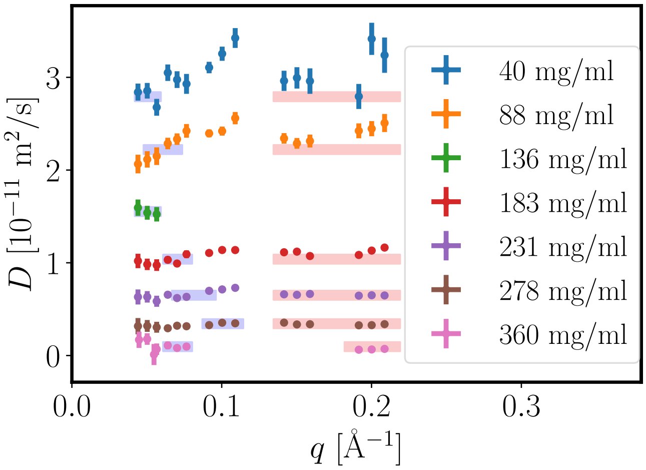

FIGURE 11 Diffusion function $D(q)$ obtained from NSE from the initial decay of the intermediate scattering function $I(q, t)$. The colored boxes indicate the q-ranges used to calculate the cage diffusion coefficient $D(q^*)$ around the principal peak position q^* of the structure factor and the high-q limit of $D(q)$, as reported in Fig. 12. To see this figure in color, go online.

The short-time self-diffusion of sticky hard spheres has also been predicted to be (49)

$$D_s/D_0 = 1 - (1.8315 + 0.295 / \tau)\phi = 1 - 2.213\phi \quad (21)$$

using the stickiness parameter $\tau = 0.774$. In Fig. 12, we display this relation with an effective volume fraction of $\phi = c\nu_{\text{eff}}$ with the voluminosity $\nu_{\text{eff}} = 1.7$ mL/g and obtain reasonable agreement when considering the additional contributions of rotations and internal motions. We have restricted the theoretical prediction to volume fractions below 0.3 because the prediction only covers the linear term of the series expansion.

DISCUSSION

In summary, our comprehensive characterization of crowding effects in β_H crystallin solutions using light, neutron, and x-ray scattering techniques, as well as tracer-based microrheology, supports the basic model picture of a solution of polydisperse hard spheres with additional mild attractive interactions and a corrugated surface. Although structural aspects could not be reproduced in full quantitative detail but follow the expected trends, we obtain a very good description of dynamics from the local nanometer scale of cage diffusion to a mesoscopic micrometer scale of gradient diffusion to the macroscopic scale of viscosity. Cumulant analysis of DLS data in the dilute limit evidences a considerable polydispersity, with a z-averaged hydrodynamic radius of 6.5 nm. No changes in the gradient diffusion via DLS were observed in the virial regime, which implies a weak additional attraction to excluded volume with an overall reduced virial coefficient of $B_2/B_2^{(HS)} \approx 0.677$. The viscosity from microrheology and the slow relaxation mode in DLS at higher concentrations point toward a power-law-like divergence of the viscosity due to dynamical arrest at a protein concentration around 360 mg/mL. The form factor from SAXS on dilute solutions is consistent with the model. The experimental structure factors obtained from SAXS show a surprisingly low and broad correlation peak, which could be qualitatively reproduced in simulations of polydisperse spheres with mild attraction. Based on dynamical arrest and hydrodynamic radius, we estimate the effective voluminosity of β_H crystallin as $\nu_{\text{eff}} = 1.7$ mL/g. Finally, the cage diffusion and local diffusion at high q from NSE characterizing motions on the scale of individual proteins are consistent with a theoretical prediction for weakly attractive hard spheres with $B_2/B_2^{(HS)} \approx 0.677$.

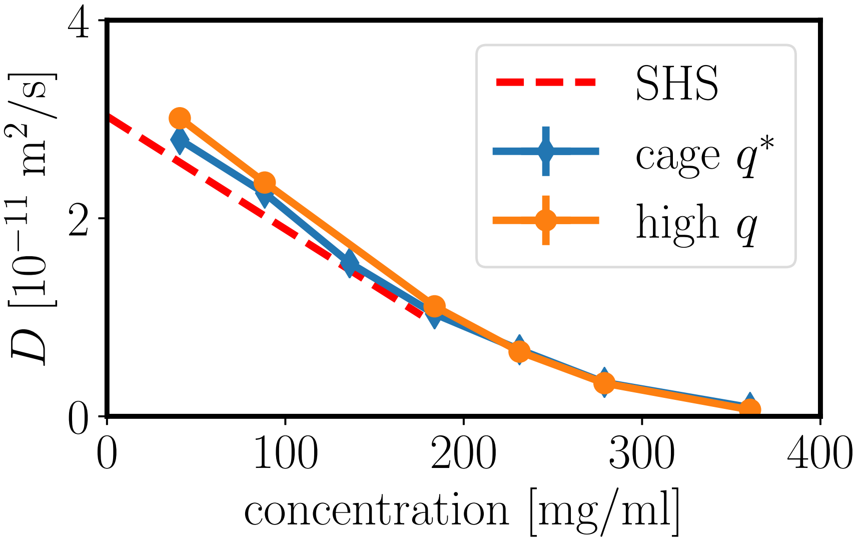

FIGURE 12 Crowding dependence of the diffusion coefficient extracted at high $q \approx 0.18$–0.22 Å^{-1} (*orange*) and around the principal peak position q^* (cage diffusion, *blue*). We further show the theoretical prediction for short-time self-diffusion for a sticky hard sphere (SHS, *red dashed*) with $D_0 = 3.03 \times 10^{-11}$ m^2/s and stickiness parameter $\tau = 0.774$ as obtained from the previous DLS results, assuming a voluminosity of $\nu = 1.7$ mL/g. The values for both self- and cage diffusion clearly exceed the theoretical prediction, pointing toward contributions of internal and rotational dynamics, at least at lower concentrations. Experimental error bars are smaller than the symbol size. To see this figure in color, go online.

The good agreement of a simple colloidal model with experimental results on a complex multisubunit protein supports the concept to exploit colloid theory to understand crowding effects in biological systems. Comparing the obtained results on β_H crystallin with results on other crystallin proteins, we observe a comparable pathway toward dynamical arrest, which is a promising topic for future investigations both regarding their physicochemical causes and their potential implications for the biological function of the eye.

Regarding the physical picture behind the slow relaxation times and increase in viscosity (Fig. 7), it is important to stress that these observations are not linked to the formation of large aggregates, as is visible in the significant and monotonic decay of the structure factor toward low q (Figs. 8 and 9 *a*) and the macroscopic observation that none of the samples were turbid.

We remark that the colloidal model used in this study follows a clear multiscale strategy to study and understand effects on a thermodynamic and structurally coarse level by coarse-grained models such as a polydisperse colloid system for a more complex multisubunit protein. On the one hand, small disagreements in the structure factor and compressibility are thus not surprising. On the other hand, the good agreement of dynamics for such a complex protein is particularly promising in light of characterization of cellular processes in which diffusion and density relaxations play important roles for kinetics and assembly.

This approach does not imply that other effects of more molecular detail are not relevant, but rather opens future opportunities to link molecular details from a more biochemical characterization to the overall physicochemical behavior of protein solutions in a bottom-up way by, e.g., identifying the fundamental determinants governing the parameter of the successful coarse-grained model.

In the context of linking model parameters back to the molecular details, the only mild attraction appears surprising at first sight; monomers of β- and γ_B crystallins share an overall similar structure, and γ_B crystallin shows significant attractions that lead to liquid-liquid phase separation. Reconsidering the situation, β-crystallin monomers have in fact most likely even more attractive interaction but saturate this attraction via the formation of stable oligomeric states. These multisubunit complexes, the β_H crystallin, consequentially do not show strong remaining interparticle attraction. Thus, the very comparable molecular details lead to vastly different macroscopic thermodynamic phase behavior; γ-crystallin shows liquid-liquid phase separation that governs the dynamics in the sample over a broad concentration and temperature range, and β-crystallin assembles into comparably inert complexes that show the simple phase behavior of hard spheres.

Despite this deviation in thermodynamic properties, a strikingly similar behavior is obtained for the dynamical arrest for crystallin proteins. α- and β_H crystallin consist of multisubunit complexes and show dynamical arrest consistent with hard-sphere predictions (28). Even for γ_B crystallin, the arrest line is temperature independent (20,60) and thus not driven by the attraction. The most probable explanation is the formation of transient clusters that—analogously to the multisubunit complexes of β_H crystallin—saturate the intermonomer attraction and then arrest as hard-sphere-like particles. Thus, dynamical arrest in crystallin solutions appears to be driven by multisubunit complexes that are stable for α- and β_H crystallin and transient for γ_B crystallin. Strikingly, even the protein concentrations of dynamical arrest are similar around 340–380 mg/mL, which implies similar voluminosity values for all crystallin complexes around 1.7 mL/g. Given the specific volume of 0.71–0.74 mL/g, all crystallin proteins occur in complexes that contain clearly more water than amino acid material. Whether or not this remarkably similar behavior is recovered in crystallin mixtures and thus is of importance for the eye lens fluid is an important question for future studies.

SUPPORTING MATERIAL

Supporting Material can be found online at https://doi.org/10.1016/j.bpj.2020.10.035.

AUTHOR CONTRIBUTIONS

A.S. and P.S. designed the research. A.G., S.B., L.C.-D., T.G., N.S.-G., M.O.-R., B.F., P.S., and A.S. performed experiments. F.R.-R. and E.Z. performed simulations. All authors analyzed the data. F.R.-R., A.G., P.S., and A.S. wrote the article with input and help from the other authors.

ACKNOWLEDGMENTS

We thank Najet Mahmoudi for her help with sample preparation.

We gratefully acknowledge financial support from the Knut and Alice Wallenberg Foundation (project grant KAW 2014.0052), the Swedish Research Council (VR; grants 2009-6794, 2016-03301 and 2018-04627), the Faculty of Science at Lund University, the European Research Council (ERC-339678-COMPASS), the Royal Physiographic Society in Lund, and the Per-Eric and Ulla Schyberg's Foundation. This work is based on experiments performed at the IN15 at the Institute Laue-Langevin, Grenoble, France (61).

SUPPORTING CITATIONS

References (62,63) appear in the Supporting Material.

REFERENCES

1. Benedek, G. B. 1997. Cataract as a protein condensation disease: the Proctor Lecture. *Invest. Ophthalmol. Vis. Sci.* 38:1911–1921.
2. Glasser, A., and M. C. Campbell. 1999. Biometric, optical and physical changes in the isolated human crystalline lens with age in relation to presbyopia. *Vision Res.* 39:1991–2015.
3. Petrash, J. M. 2013. Aging and age-related diseases of the ocular lens and vitreous body. *Invest. Ophth. Vis. Sci.* 54:ORSF54-9.

4. Zimmerman, S. B., and A. P. Minton. 1993. Macromolecular crowding: biochemical, biophysical, and physiological consequences. *Annu. Rev. Biophys. Biomol. Struct.* 22:27–65.

5. Dix, J. A., and A. S. Verkman. 2008. Crowding effects on diffusion in solutions and cells. *Annu. Rev. Biophys.* 37:247–263.

6. Ellis, R. J. 2001. Macromolecular crowding: obvious but underappreciated. *Trends Biochem. Sci.* 26:597–604.

7. Johnston, K. P., J. A. Maynard, …, K. J. Kaczorowski. 2012. Concentrated dispersions of equilibrium protein nanoclusters that reversibly dissociate into active monomers. *ACS Nano.* 6:1357–1369.

8. Skar-Gislinge, N., M. Ronti, …, A. Stradner. 2019. A colloid approach to self-assembling antibodies. *Mol. Pharm.* 16:2394–2404.

9. Roos, M., M. Ott, …, K. Saalwächter. 2016. Coupling and decoupling of rotational and translational diffusion of proteins under crowding conditions. *J. Am. Chem. Soc.* 138:10365–10372.

10. Ando, T., and J. Skolnick. 2010. Crowding and hydrodynamic interactions likely dominate in vivo macromolecular motion. *Proc. Natl. Acad. Sci. USA.* 107:18457–18462.

11. Roosen-Runge, F., M. Hennig, …, F. Schreiber. 2011. Protein self-diffusion in crowded solutions. *Proc. Natl. Acad. Sci. USA.* 108:11815–11820.

12. Grimaldo, M., H. Lopez, …, F. Schreiber. 2019. Protein short-time diffusion in a naturally crowded environment. *J. Phys. Chem. Lett.* 10:1709–1715.

13. Grimaldo, M., F. Roosen-Runge, …, T. Seydel. 2019. Dynamics of proteins in solution. *Q. Rev. Biophys.* 52:1–63.

14. Stradner, A., and P. Schurtenberger. 2020. Potential and limits of a colloid approach to protein solutions. *Soft Matter.* 16:307–323.

15. Longeville, S., W. Doster, and G. Kali. 2003. Myoglobin in crowded solutions: structure and diffusion. *Chem. Phys.* 292:413–424.

16. Doster, W., and S. Longeville. 2007. Microscopic diffusion and hydrodynamic interactions of hemoglobin in red blood cells. *Biophys. J.* 93:1360–1368.

17. Häussler, W. 2008. Neutron spin echo studies on ferritin: free-particle diffusion and interacting solutions. *Eur. Biophys. J.* 37:563–571.

18. Cardinaux, F., E. Zaccarelli, …, P. Schurtenberger. 2011. Cluster-driven dynamical arrest in concentrated lysozyme solutions. *J. Phys. Chem. B.* 115:7227–7237.

19. Godfrin, P. D., S. D. Hudson, …, Y. Liu. 2015. Short-time glassy dynamics in viscous protein solutions with competing interactions. *Phys. Rev. Lett.* 115:228302.

20. Bucciarelli, S., L. Casal-Dujat, …, A. Stradner. 2015. Unusual dynamics of concentration fluctuations in solutions of weakly attractive globular proteins. *J. Phys. Chem. Lett.* 6:4470–4474.

21. Bucciarelli, S., J. S. Myung, …, A. Stradner. 2016. Dramatic influence of patchy attractions on short-time protein diffusion under crowded conditions. *Sci. Adv.* 2:e1601432.

22. Heinen, M., F. Zanini, …, G. Nägele. 2012. Viscosity and diffusion: crowding and salt effects in protein solutions. *Soft Matter.* 8:1404–1419.

23. Grimaldo, M., F. Roosen-Runge, …, F. Schreiber. 2014. Diffusion and dynamics of γ-globulin in crowded aqueous solutions. *J. Phys. Chem. B.* 118:7203–7209.

24. Augusteyn, R., and A. Stevens. 1998. Macromolecular structure of the eye lens. *Prog. Polym. Sci.* 23:375–413.

25. Bloemendal, H., W. de Jong, …, A. Tardieu. 2004. Ageing and vision: structure, stability and function of lens crystallins. *Prog. Biophys. Mol. Biol.* 86:407–485.

26. Braun, N., M. Zacharias, …, S. Weinkauf. 2011. Multiple molecular architectures of the eye lens chaperone αB-crystallin elucidated by a triple hybrid approach. *Proc. Natl. Acad. Sci. USA.* 108:20491–20496.

27. Finet, S., and A. Tardieu. 2001. α-crystallin interaction forces studied by small angle X-ray scattering and numerical simulations. *J. Cryst. Growth.* 232:40–49.

28. Foffi, G., G. Savin, …, P. Schurtenberger. 2014. Hard sphere-like glass transition in eye lens α-crystallin solutions. *Proc. Natl. Acad. Sci. USA.* 111:16748–16753.

29. Vérétout, F., M. Delaye, and A. Tardieu. 1989. Molecular basis of eye lens transparency. Osmotic pressure and X-ray analysis of α-crystallin solutions. *J. Mol. Biol.* 205:713–728.

30. Thomson, J. A., P. Schurtenberger, …, G. B. Benedek. 1987. Binary liquid phase separation and critical phenomena in a protein/water solution. *Proc. Natl. Acad. Sci. USA.* 84:7079–7083.

31. Schurtenberger, P., R. A. Chamberlin, …, G. B. Benedek. 1989. Observation of critical phenomena in a protein-water solution. *Phys. Rev. Lett.* 63:2064–2067.

32. Tardieu, A., F. Vérétout, …, C. Slingsby. 1992. Protein interactions in the calf eye lens: interactions between β-crystallins are repulsive whereas in γ-crystallins they are attractive. *Eur. Biophys. J.* 21:1–12.

33. Myung, J. S., F. Roosen-Runge, …, A. Stradner. 2018. Weak shape anisotropy leads to a nonmonotonic contribution to crowding, impacting protein dynamics under physiologically relevant conditions. *J. Phys. Chem. B.* 122:12396–12402.

34. Bateman, O. A., and C. Slingsby. 1992. Structural studies on β H-crystallin from bovine eye lens. *Exp. Eye Res.* 55:127–133.

35. Liu, H., S. K. Kumar, and F. Sciortino. 2007. Vapor-liquid coexistence of patchy models: relevance to protein phase behavior. *J. Chem. Phys.* 127:084902.

36. Berbers, G. A. M., O. C. Boerman, …, W. W. de Jong. 1982. Primary gene products of bovine β-crystallin and reassociation behavior of its aggregates. *Eur. J. Biochem.* 128:495–502.

37. Bindels, J. G., A. Koppers, and H. J. Hoenders. 1981. Structural aspects of bovine β-crystallins: physical characterization including dissociation-association behavior. *Exp. Eye Res.* 33:333–343.

38. Garting, T., and A. Stradner. 2018. Optical microrheology of protein solutions using tailored nanoparticles. *Small.* 14:e1801548.

39. Thurston, G. M. 2006. Liquid-liquid phase separation and static light scattering of concentrated ternary mixtures of bovine alpha and gammaB crystallins. *J. Chem. Phys.* 124:134909.

40. Moitzi, C., R. Vavrin, …, P. Schurtenberger. 2009. A new instrument for time-resolved static and dynamic light-scattering experiments in turbid media. *J. Colloid Interface Sci.* 336:565–574.

41. Mailer, A. G., P. S. Clegg, and P. N. Pusey. 2015. Particle sizing by dynamic light scattering: non-linear cumulant analysis. *J. Phys. Condens. Matter.* 27:145102.

42. Garting, T., and A. Stradner. 2019. Synthesis and application of PEGylated tracer particles for measuring protein solution viscosities using Dynamic Light Scattering-based microrheology. *Colloids Surf. B Biointerfaces.* 181:516–523.

43. Block, I. D., and F. Scheffold. 2010. Modulated 3D cross-correlation light scattering: improving turbid sample characterization. *Rev. Sci. Instrum.* 81:123107.

44. Rapaport, D. C. 2004. The Art of Molecular Dynamics Simulation. Cambridge University Press, Cambridge, UK.

45. Zaccarelli, E., C. Valeriani, …, P. N. Pusey. 2009. Crystallization of hard-sphere glasses. *Phys. Rev. Lett.* 103:135704.

46. Koppel, D. E. 1972. Analysis of macromolecular polydispersity in intensity correlation spectroscopy: the method of cumulants. *J. Chem. Phys.* 57:4814–4820.

47. Perrin, F. 1934. Mouvement Brownien d'un ellipsoide (I). Dispersion dielectrique pour des molecules ellipsoidales. *J. Phys. Radium.* 7:497–511.

48. Brunetti, S., D. Roux, …, P. Bothorel. 1983. Micellar interactions in water-in-oil microemulsions. 2. Light scattering determination of the second virial coefficient. *J. Phys. Chem.* 87:1028–1034.

49. Cichocki, B., and B. U. Felderhof. 1990. Diffusion coefficients and effective viscosity of suspensions of sticky hard spheres with hydrodynamic interactions. *J. Chem. Phys.* 93:4427–4432.

50. De Kruif, C. G., P. W. Rouw, …, R. P. May. 1989. Adhesive hard-sphere colloidal dispersions. A small-angle neutron-scattering study of stickiness and the structure factor. *Langmuir.* 5:422–428.
51. Braun, M. K., M. Wolf, …, F. Schreiber. 2017. Strong isotope effects on effective interactions and phase behavior in protein solutions in the presence of multivalent ions. *J. Phys. Chem. B.* 121:1731–1739.
52. Bucciarelli, S., N. Mahmoudi, …, A. Stradner. 2016. Extended law of corresponding states applied to solvent isotope effect on a globular protein. *J. Phys. Chem. Lett.* 7:1610–1615.
53. Goryunov, A. S. 2006. H/D isotope effects on protein hydration and interaction in solution. *Gen. Physiol. Biophys.* 25:303–311.
54. Puertas, A. M., M. Fuchs, and M. E. Cates. 2003. Simulation study of nonergodicity transitions: gelation in colloidal systems with short-range attractions. *Phys. Rev. E Stat. Nonlin. Soft Matter Phys.* 67:031406.
55. Puertas, A. M., C. De Michele, …, E. Zaccarelli. 2007. Viscoelasticity and Stokes-Einstein relation in repulsive and attractive colloidal glasses. *J. Chem. Phys.* 127:144906.
56. Griffith, W. L., R. Triolo, and A. L. Compere. 1987. Analytical scattering function of a polydisperse Percus-Yevick fluid with Schulz- (Gamma -) distributed diameters. *Phys. Rev. A Gen. Phys.* 35:2200–2206.
57. Sharma, R., and K. Sharma. 1977. The structure factor and the transport properties of dense fluids having molecules with square well potential, a possible generalization. *Physica A.* 89:213–218.
58. Nägele, G. 1996. On the dynamics and structure of charge-stabilized suspensions. *Phys. Rep.* 272:215–372.
59. Biehl, R., M. Monkenbusch, and D. Richter. 2011. Exploring internal protein dynamics by neutron spin echo spectroscopy. *Soft Matter.* 7:1299–1307.
60. Bergman, M. 2019. On the phase behaviour of soft matter: understanding complex interactions via quantitative imaging. Doctoral dissertation. Lund University.
61. Stradner, A., S. Bucciarelli, …, P. Schurtenberger. Looking at the diffusion in crowded eye lens protein mixtures using a colloid-based approach: Institut Laue-Langevin (ILL). doi: 10.5291/ILL-DATA.9–13–474. Available from: https://doi.ill.fr/10.5291/ILL-DATA.9-13-474.
62. Pauw, B. R., J. S. Pedersen, …, B. B. Iversen. 2013. Improvements and considerations for size distribution retrieval from small-angle scattering data by Monte Carlo methods. *J. Appl. Cryst.* 46:365–371.
63. Bressler, I., B. R. Pauw, and A. F. Thünemann. 2015. *McSAS*: software for the retrieval of model parameter distributions from scattering patterns. *J. Appl. Cryst.* 48:962–969.

Biophysical Journal, Volume *119*

Supplemental Information

Crowding in the Eye Lens: Modeling the Multisubunit Protein β-Crystallin with a Colloidal Approach

Felix Roosen-Runge, Alessandro Gulotta, Saskia Bucciarelli, Lucía Casal-Dujat, Tommy Garting, Nicholas Skar-Gislinge, Marc Obiols-Rabasa, Bela Farago, Emanuela Zaccarelli, Peter Schurtenberger, and Anna Stradner

Biophysical *Journal*
Supporting Information

Supporting information: Crowding in the eye lens: modeling the multi-subunit protein β crystallin with a colloidal approach

Felix Roosen-Runge[1,*], Alessandro Gulotta[1], Saskia Bucciarelli[1], Lucía Casal-Dujat[1], Tommy Garting[1], Nicholas Skar-Gislinge[1], Marc Obiols-Rabasa[1], Bela Farago[2], Emanuela Zaccarelli[3,4], Peter Schurtenberger[1], and Anna Stradner[1,*]

[1]Division of Physical Chemistry, Lund University, Naturvetarvägen 14, 22100 Lund, Sweden
[2]Institut Laue-Langevin, 71 avenue des Martyrs, 38042 Grenoble, France
[3]Institute for Complex Systems, National Research Council (ISC-CNR), Uos Sapienza, Piazzale Aldo Moro 5, 00185 Rome, Italy
[4]Department of Physics, Sapienza Università di Roma, Piazzale Aldo Moro 1, 00185 Rome, Italy
[*]Correspondence: felix.roosen-runge@mau.se, anna.stradner@fkem1.lu.se

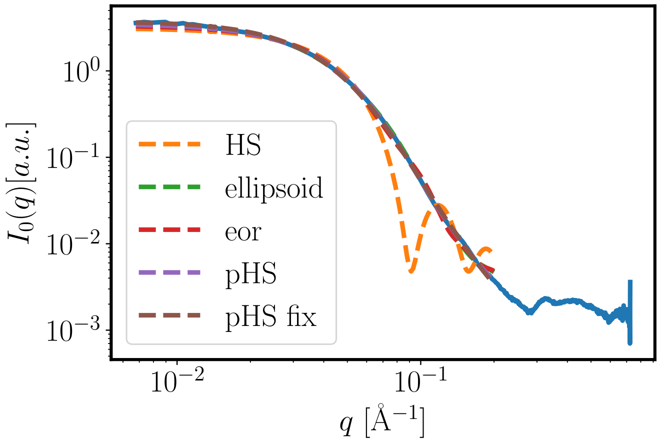

Figure S1: Different model fits (dashed lines) to the SAXS form factor of β_H crystallin (full line). While the hard sphere form factor (HS) cannot fit the data at all, form factors for a triaxial ellipsoid, an ellipsoid of revolution (eor) and polydisperse hard sphere systems provide reasonable fits. Given the evidence for polydisperse solutions, and the unfavorable R_h/R_g ratio for ellipsoids, we focused on the polydisperse hard sphere systems, and used both a fit with free parameter (pHS) and one with fixed radius of gyration (pHS fix; reported in main article).

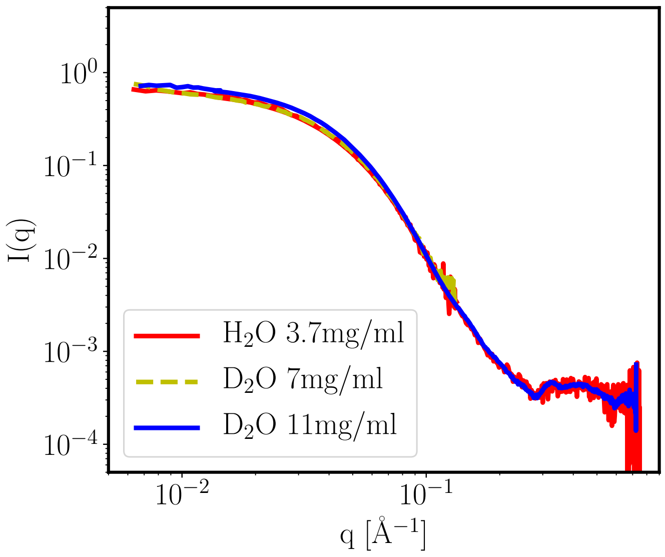

Figure S2: Form factor of β_H crystallin at different low protein concentrations measured by SAXS. All profiles in H_2O and D_2O show consistent results.

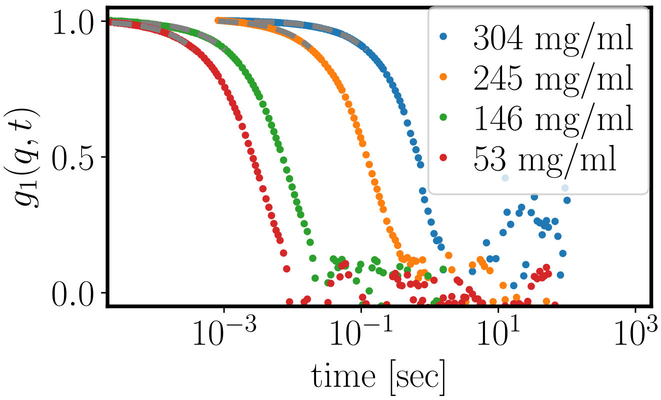

Figure S3: Exemplary correlation function from microrheology for tracer particles in concentrated β_H crystallin solutions. From the initial slope, the diffusion coefficient of the large tracers is calculated, which is directly related to the viscosity of the protein solution.

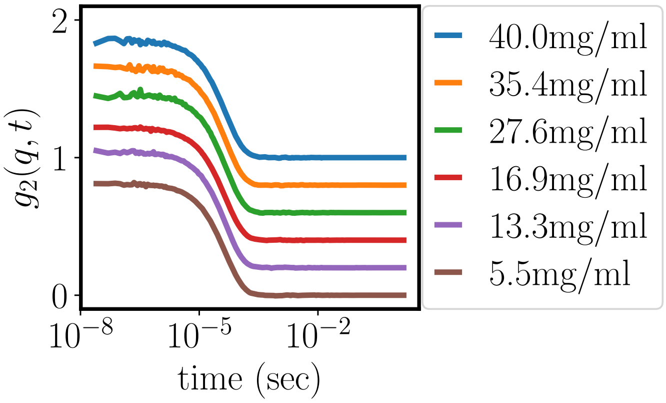

Figure S4: Exemplary correlation functions of β_H crystallin from DLS at a scattering angle 90° for lower concentrations. The data were shifted by 0.2 for better visibility, as the relaxation time is constant throughout the concentration range.

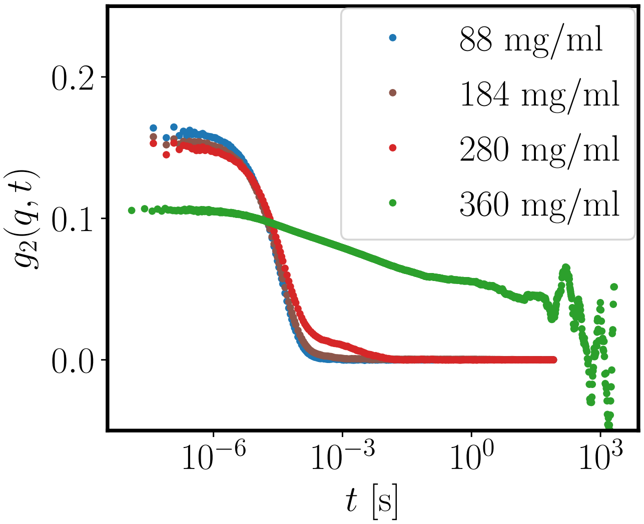

Figure S5: Autocorrelation functions of β_H crystallin from DLS at a scattering angle 90° for higher concentrations at q = 0.22 nm^{1}. While the three lower concentrations show reasonable profiles, the undefined baseline and the low intercept indicate non-ergodic sample properties for the highest protein concentration of 360 mg/ml. This profile was thus not fitted, but rather indicated a concentration where dynamical arrest has already set in.

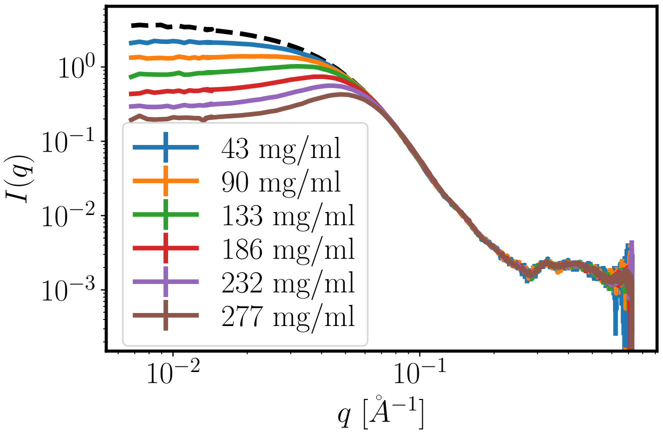

Figure S6: Normalized scattering intensities from SAXS for a concentration series of β_H crystallin. The form factor (dashed black line, 11 mg/ml) is used to calculate the experimental structure factor reported in the main article.

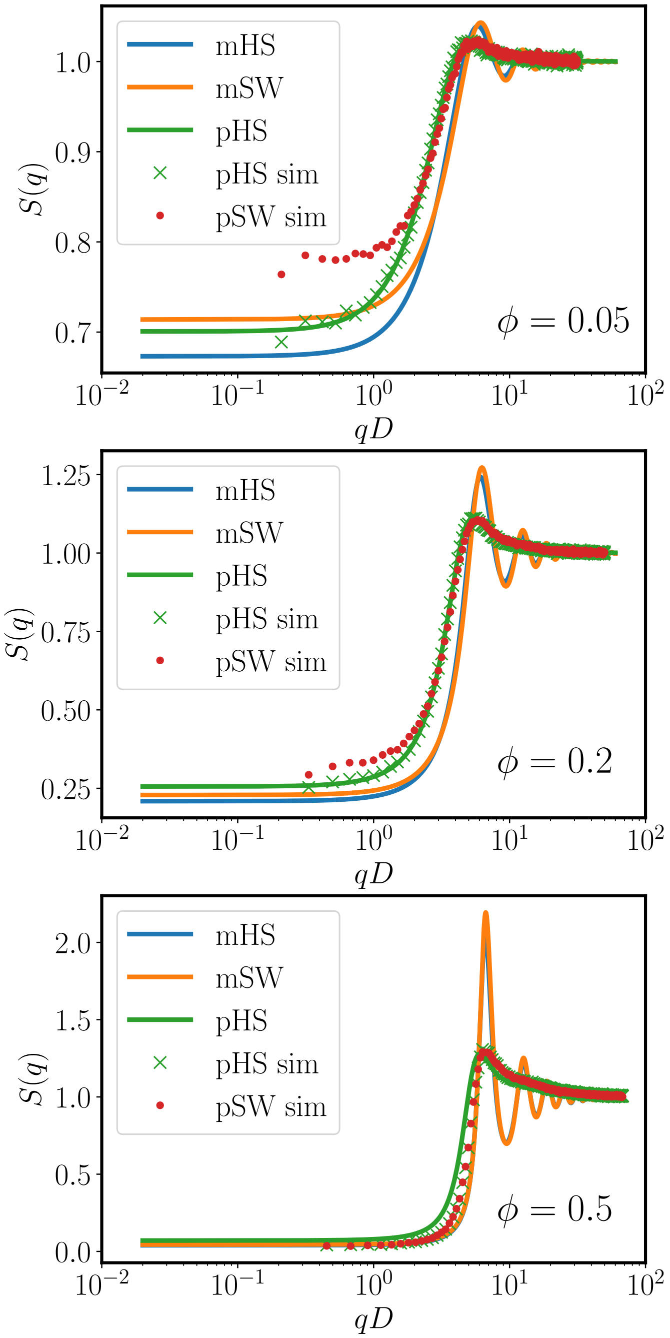

Figure S7: Effects of polydispersity and attraction on $S(q)$. The lines correspond to theoretical calculations of structure factors for monodisperse hard spheres (mHS), polydisperse hard spheres (pHS) and monodisperse hard spheres with additional square-well attraction (mSW). The symbols indicate simulation results of polydisperse hard spheres with and without additional square-well attraction.

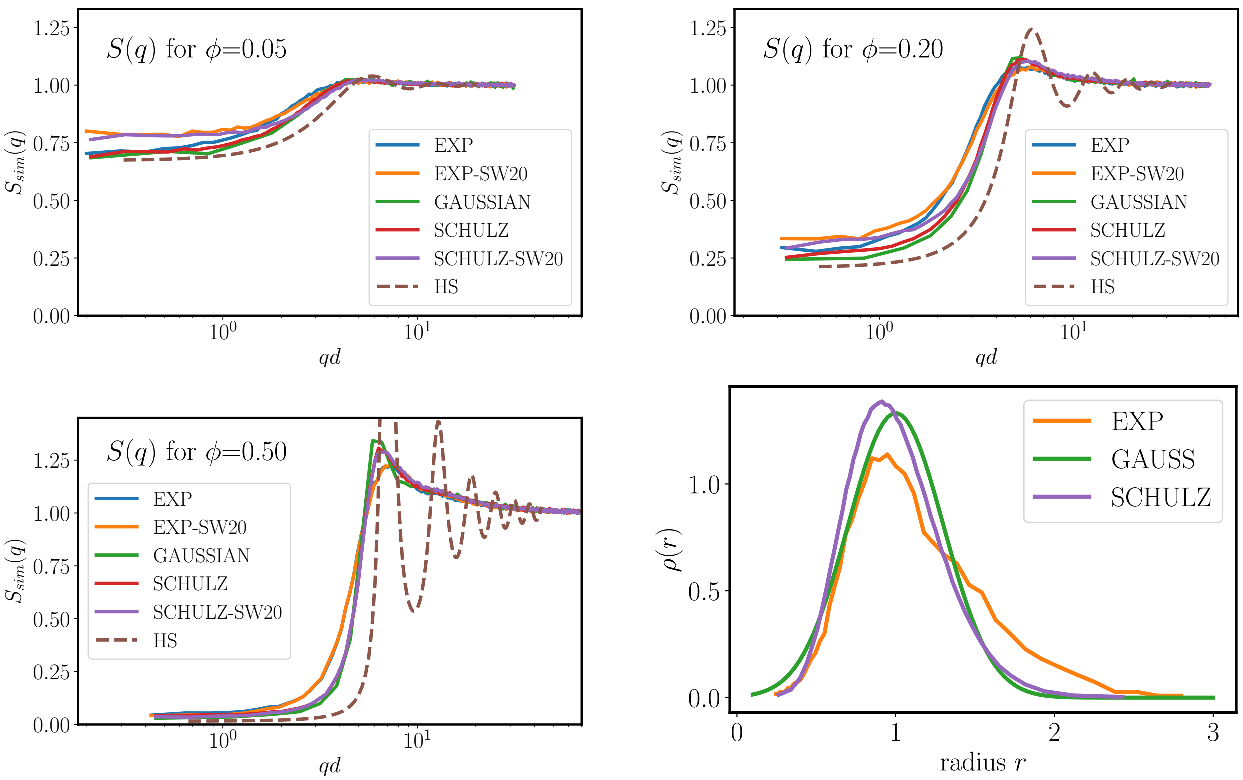

Figure S8: Effects of different size distributions (lower right) on the effective structure factor $S(q)$. The lines correspond to simulation of hard spheres with an exploratory size distribution (EXP), a Gaussian size distribution (GAUSS) and a discretized Schulz size distribution (SCHULZ), all with a normalized standard deviation of $\sigma^* = 0.3$. The discretized Schulz and the exploratory distribution were also simulated with an additional square-well attraction with a range of 0.2 of the diameter d and a depth of 0.289 $k_B T$ (SW20). In addition, the structure factor of a monodisperse hard sphere is shown (HS).

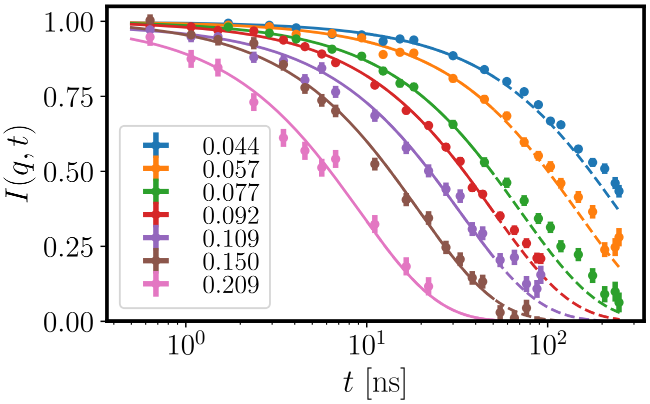

Figure S9: Intermediate scattering function $I(q,t)$ from neutron spin echo spectroscopy for β_H crystallin in D_2O phosphate buffer at a concentration of 88 mg/ml. The legend specifies the q value in Å^{-1}.

Exploratory size distribution

In addition to the methods used in the main text, we explored the use of inverse Monte Carlo methods for fitting of the form factor. We used the program package mcSAS (1, 2) which determines a distribution of hard spheres based on minimization of the experimental residuals, and additional regularization parameters to smoothen the distribution. As basic result, we obtained a distribution with a functional form very close to the Schulz distribution (see Fig. S8 lower right).

REFERENCES

1. Pauw, B. R., J. S. Pedersen, S. Tardif, M. Takata, and B. B. Iversen, 2013. Improvements and considerations for size distribution retrieval from small-angle scattering data by Monte Carlo methods. *Journal of Applied Crystallography* 46:365–371.

2. Bressler, I., B. R. Pauw, and A. F. Thünemann, 2015. *McSAS*: software for the retrieval of model parameter distributions from scattering patterns. *Journal of Applied Crystallography* 48:962–969.
